# Differential NtcA Responsiveness to 2-Oxoglutarate Underlies the Diversity of C/N Balance Regulation in *Prochlorococcus*

**DOI:** 10.3389/fmicb.2017.02641

**Published:** 2018-01-09

**Authors:** María A. Domínguez-Martín, Antonio López-Lozano, Rafael Clavería-Gimeno, Adrián Velázquez-Campoy, Gerald Seidel, Andreas Burkovski, Jesús Díez, José M. García-Fernández

**Affiliations:** ^1^Departamento de Bioquímica y Biología Molecular, Campus de Excelencia Internacional Agroalimentario CeiA3, Universidad de Córdoba, Córdoba, Spain; ^2^Institute of Biocomputation and Physics of Complex Systems (BIFI), Joint Units BIFI-IQFR-CSIC and GBsC-BIFI-CSIC, Universidad de Zaragoza, Zaragoza, Spain; ^3^Aragon Institute for Health Research (IIS Aragon), Zaragoza, Spain; ^4^Instituto Aragonés de Ciencias de la Salud, Zaragoza, Spain; ^5^Centro de Investigación Biomédica en Red en el Área Temática de Enfermedades Hepáticas y Digestivas, Barcelona, Spain; ^6^Fundación ARAID, Gobierno de Aragón, Zaragoza, Spain; ^7^Professur für Mikrobiologie, Department Biologie, Friedrich-Alexander-Universität Erlangen-Nürnberg, Erlangen, Germany

**Keywords:** *Prochlorococcus*, cyanobacteria, 2-oxoglutarate, streamlined regulation, C/N balance

## Abstract

Previous studies showed differences in the regulatory response to C/N balance in *Prochlorococcus* with respect to other cyanobacteria, but no information was available about its causes, or the ecological advantages conferred to thrive in oligotrophic environments. We addressed the changes in key enzymes (glutamine synthetase, isocitrate dehydrogenase) and the *ntcA* gene (the global nitrogen regulator) involved in C/N metabolism and its regulation, in three model *Prochlorococcus* strains: MED4, SS120, and MIT9313. We observed a remarkable level of diversity in their response to azaserine, a glutamate synthase inhibitor which increases the concentration of the key metabolite 2-oxoglutarate, used to sense the C/N balance by cyanobacteria. Besides, we studied the binding between the global nitrogen regulator (NtcA) and the promoter of the *glnA* gene in the same *Prochlorococcus* strains, and its dependence on the 2-oxoglutarate concentration, by using isothermal titration calorimetry, surface plasmon resonance, and electrophoretic mobility shift. Our results show a reduction in the responsiveness of NtcA to 2-oxoglutarate in *Prochlorococcus*, especially in the MED4 and SS120 strains. This suggests a trend to streamline the regulation of C/N metabolism in late-branching *Prochlorococcus* strains (MED4 and SS120), in adaptation to the rather stable conditions found in the oligotrophic ocean gyres where this microorganism is most abundant.

## Introduction

The extraordinary abundance of *Prochlorococcus* in the oligotrophic areas of the oceans has been reported by different studies since its discovery (Chisholm et al., 1988), and it is considered that *Prochlorococcus* contributes significantly to the global primary production (Goericke and Welschmeyer, 1993; Liu et al., 1997; Casey et al., 2007), conferring this organism an outstanding importance in marine ecology (Biller et al., 2015). Recent reports suggest it might become even more widespread due to global warming in the near future (Flombaum et al., 2013). Different explanations have been proposed for the abundance of *Prochlorococcus*, including the very small size of the cells, the high surface/volume ratio, or the streamlining of the genome (and consequently of the metabolic pathways), leading to low energetic requirements for survival and cell division (Partensky et al., 1999; Giovannoni et al., 2014; Biller et al., 2015).

Adaptation to oligotrophic oceans involves the development of adaptive mechanisms to cope with the availability of several key elements, which are scarce in those regions. Nitrogen is one of them. This fact has led to different strategies to optimize its utilization in marine cyanobacteria, providing enough nitrogen for growth with an affordable energetic expense, such as the use of specific sets of N-assimilating genes depending on the strain (López-Lozano et al., 2002; Moore et al., 2002; Dufresne et al., 2003, 2008; Palenik et al., 2003; Rocap et al., 2003; García-Fernández et al., 2004; Scanlan et al., 2009; Berube et al., 2014; Biller et al., 2014).

The cyanobacterial regulatory mechanisms to control the C/N balance are present in *Prochlorococcus*, but showing some striking variations with respect to freshwater strains: despite possessing the genes involved in standard carbon/nitrogen regulation in cyanobacteria (namely *ntcA, glnB*, and *pipX* encoding, respectively, NtcA, P_II_, and PipX), they do not seem to work as previously described (Lindell et al., 2002; Palinska et al., 2002; García-Fernández et al., 2004; López-Lozano et al., 2009; Domínguez-Martín et al., 2014). Besides, the regulation of enzymes such as glutamine synthetase (GS) (El Alaoui et al., 2001, 2003; Gómez-Baena et al., 2001) and isocitrate dehydrogenase (ICDH) (López-Lozano et al., 2009; Domínguez-Martín et al., 2014) is clearly different from their counterparts in other cyanobacteria, showing a lack of response to nitrogen starvation or darkness. Furthermore, comparative transcriptomic studies have shown clear changes in two of the main model *Prochlorococcus* strains, such as MED4 and MIT9313, suggesting that they integrate N and C metabolism in fundamentally different ways (Tolonen et al., 2006). However, the molecular underpinnings of these changes have not been studied thus far.

We studied the role of 2-oxoglutarate (2OG) in the control of the C/N balance in *Prochlorococcus*, in order to check whether there are differences with respect to other model cyanobacteria (Forchhammer and Tandeau De Marsac, 1994; Vazquez-Bermudez et al., 2002; Muro-Pastor and Florencio, 2003; Flores and Herrero, 2005; Muro-Pastor et al., 2005; Luque and Forchhammer, 2008). Besides, we addressed the regulatory differences between *Prochlorococcus* strains by studying the enzymes glutamine synthetase (GS) and isocitrate dehydrogenase (ICDH) and also the interaction between the NtcA protein and the promoter of the *glnA* gene (encoding GS). To this goal, we measured enzymatic activities by spectrophotometric methods, enzyme concentrations by Western blotting, gene expression by qRT-PCR, intracellular 2OG concentration by an enzymatic method and the interaction of NtcA with *glnA* promoter DNA by isothermal titration calorimetry (ITC), surface plasmon resonance (SPR) and electrophoretic mobility shift assay (EMSA).

Azaserine is an inhibitor of glutamate synthase (GOGAT) (Pinkus, 1977), the enzyme that together with GS compose the main pathway for nitrogen assimilation in cyanobacteria. We have previously shown that azaserine induces clear changes in enzyme activities related to nitrogen metabolism of several *Prochlorococcus* strains (El Alaoui et al., 2001; López-Lozano et al., 2009; Rangel et al., 2009; Domínguez-Martín et al., 2014). Furthermore, we have recently shown that azaserine addition induces an increase in the concentration of 2OG in *Prochlorococcus* SS120, mimicking the effect of nitrogen starvation (Domínguez-Martín et al., 2017); this leads to extensive changes in the proteome of this strain (Domínguez-Martín et al., 2017). Hence we decided to study the effects of azaserine addition to cultures of different *Prochlorococcus* strains.

In this study we compared three different strains: MED4, representative member of ecotypes adapted to live near the ocean surface, and MIT9313 and SS120 belonging to ecotypes adapted to live at depth. Despite both strains living at depth, MIT9313 and SS120 are very different in evolutionary terms, as MIT9313 was early branching in the *Prochlorococcus* phylogenetic tree (Biller et al., 2014). Our goal was to test if differences in the regulation of the C/N balance might be related to the different habitats of strains, and/or to their phylogeny.

## Materials and methods

### *Prochlorococcus* strains and growth conditions

*Prochlorococcus* spp. strains MED4 (high-irradiance adapted), SS120 and MIT9313 (low-irradiance adapted) were cultured as described (El Alaoui et al., 2001). Cultures were grown in a culture room set at 24°C under continuous blue irradiances: 40 μE/m^2^/s for high-light adapted ecotypes and 4 μE/m^2^/s for low-light adapted ecotypes using neon *Sylvania* F18W/154-ST *Daylight*, covered with a filter *Moonlight blue L183* from *Lee Filters*.

### Cell extracts

Cells were centrifuged at 26,000 g for 8 min at 4°C using an *Avanti J-25 Beckman* centrifuge equipped with a JA-14 rotor. After pouring most of the supernatant and carefully pipetting out the remaining medium, the pellet was directly resuspended in the corresponding volume of buffer (generally 1 mL per liter of culture). For Western blotting the proportion was 0.5 mL per liter of culture. For protein assays (enzymatic and Western blotting) the solution was 50 mM Tris-HCl pH 7.5 and for RNA analysis the pellet was resuspended in 10 mM sodium acetate (pH 4.5), 200 mM sucrose and 5 mM EDTA.

After thawing, the cell suspensions were broken in a French pressure cell (*SLM/Aminco model FA-079*) at 16,000 psi; the obtained extracts were centrifuged for 10 min at 16,900 g and 4°C in an *Eppendorf* microfuge *5418R*. The supernatant was transferred to a new tube.

### Determination of protein concentration

Protein concentration was determined using the *Bio-Rad Protein Assay* kit, according to the manufacturer instructions.

### Determination of intracellular concentration of 2OG

After thawing the samples on ice, the extracts were centrifuged for 10 min at 16,900 g and 4°C. The supernatants were used for the determination of 2OG as previously described (Domínguez-Martín et al., 2014).

### Determination of enzymatic activities

Glutamine synthetase transferase activity and NADP-isocitrate dehydrogenase activity were determined as previously described (El Alaoui et al., 2001; Domínguez-Martín et al., 2014).

### Detection of glutamine synthetase and isocitrate dehydrogenase by western blotting

Western blotting was carried out as described elsewhere (Domínguez-Martín et al., 2014), by using anti-GS and anti-ICDH antibodies raised against both enzymes from *Synechocystis* sp. PCC 6803, kindly provided by Dr. M.I. Muro-Pastor and Prof. F.J. Florencio (Instituto de Bioquímica Vegetal y Fotosíntesis, Sevilla, Spain).

### RNA extraction

RNA was isolated from 500 mL culture samples. Cells were harvested as described above. Total RNA was extracted using TRIsure RNA Isolation Reagent (Bioline) following the manufacturer's recommendations, and resuspended in 200 μL sterile MilliQ-grade water, with the addition of 133 μL 8 M LiCl, an additional precipitation step included at the end of the procedure to improve the RNA quality. RNA was treated with Rnase-free DnaseI (Ambion) according to the manufacturer instructions, and the absence of contaminating genomic DNA was assessed using a PCR control test.

### Design and production of oligonucleotides

The sequences of cyanobacterial genes were obtained from CYORF (Cyanobacteria gene annotation database, http://cyano.genome.ad.jp/). The oligonucleotides used in this work were designed using the program *Oligo v4.05* (National Biosciences Inc. Plymouth), *Primer3* (University of Massachusetts Medical School, http://bioinfo.ut.ee/primer3/) and refined manually. *Integrated DNA Technologies, Sigma*, and *Invitrogen* provided the oligonucleotides. The list of oligonucleotides used is shown in Supplementary Table 1.

### Quantitation of gene expression by QRT-PCR

qRT-PCR was performed as previously described (López-Lozano et al., 2009), by using the *rnpB* expression as internal control for quantitation. The sequences of the utilized primers are shown in Supplementary Table 1.

### Construction of a *ntcA* expression plasmid

Oligonucleotides used for *ntcA* cloning from MED4, SS120, and MIT9313 are listed in the Supplementary Table 1. To obtain the expression plasmid, *ntcA* sequences were PCR amplified with high fidelity polymerases (*Fermentas* and *Bioline*) following the manufacturers instructions. The resulting fragment was cloned into pGEM®-T (*Promega*) or pSpark® (*Canvax*). From the resulting plasmid the *Nde*I-*Bam*HI fragment containing *ntcA* was cloned into the *Nde*I-*Bam*HI site of pET-15b, giving pET-15b-*ntcA*. Each step was checked by sequencing using the services from the SCAI (Servicio Central de Apoyo a la Investigación) at the University of Córdoba.

### Overexpression and purification of recombinant NtcA from *Prochlorococcus*

Since NtcA-*glnA* promoter interaction studies were done using two different techniques (ITC and SPR), in collaboration with two different laboratories, the overexpression and purification procedures were optimized for each technique, and thus are described separately.

### Overexpression and purification of recombinant NtcA from *Prochlorococcus* sp. MED4, SS120, and MIT9313 to perform ITC determinations

*Escherichia coli* HB101 served as the host strain for construction and propagation of pGEM®-T (Promega) or pSpark® (Canvax) carrying the *ntcA* gene. Expression of recombinant NtcA (His6) proteins from *Prochlorococcus* sp. MED4, MIT9313, and SS120 were carried in *E. coli* BL21 (DE3). Strains were grown in Luria broth (LB) supplemented with filtered ampicillin (100 μg/mL). An overnight 150 mL culture was diluted in 4.5 L of LB supplemented with 100 μg/mL ampicillin and grown at 37°C to an OD_600_ of 0.5–0.6. Subsequently, heterologous expression was induced with 0.5 mM IPTG (isopropyl ß-D-1-thiogalactopyranoside) and the culture was transferred to 37°C for 4 h. Thereafter, cells were harvested at 3,800 g for 10 min at room temperature. The pellet was resuspended in buffer A (50 mM NaH_2_PO_4_, 300 mM NaCl, 60 mM Imidazole) containing lysozyme (0.3 mg/mL). Disruption of cells was carried out on ice by sonication and 30 μL of Dnase I (10 mg/mL) and 30 μL of RNase (5 mg/mL) were added and incubated on ice for 20 min. Cell debris was removed by centrifugation at 48,000 g (JA-20 rotor) for 40 min at 4°C and the supernatant was collected. The purification was carried out with a Pierce Centrifuge Column, 10 mL (ThermoFisher Scientific), adding 4 mL of Ni-NTA agarose (ThermoFisher Scientific) according to the manufacturer instructions. All steps were performed at 4°C. The column was charged with the supernatant and subsequently washed with 4 column volumes of wash buffer (50 mM NaH_2_PO_4_, 300 mM NaCl, 80 mM Imidazole). The attached proteins were eluted by ligand exchange employing buffer B (50 mM NaH_2_PO_4_, 300 mM NaCl, 250 mM Imidazole). Fractions containing specific NtcA were visualized by coomassie-stained 12% SDS-PAGE. The buffer exchange of the eluted proteins to the buffer used for the isothermal titration calorimetry (50 mM Hepes, 350 mM NaCl, pH 7.5) was carried out with a Spectra/Por 2 Regenerated Cellulose Dialysis Membrane (Spectrum Labs) according to the manufacturer's instructions.

### Overexpression and purification of recombinant NtcA from *Prochlorococcus* sp. SS120 and MIT9313 to perform SPR determinations

*E. coli* HB101 and DH5α served as the host strain for construction and propagation of pGEM®-T (*Promega*) or pSpark® (*Canvax*) with *ntcA* gene cloned. Expression of recombinant NtcA (His6) proteins from *Prochlorococcus* MIT9313 and SS120 were carried in *E. coli* BL21 (DE3) (Supplementary Figure 1). Since the SPR studies were carried out earlier than those using ITC, at that time we focused our attention on two low-light adapted *Prochlorococcus* strains with very different evolutionary histories, as MIT9313 and SS120, and therefore this interaction was not studied by SPR in the strain MED4. Strains were grown in Luria broth (LB) supplemented with filtered ampicillin (100 μg/mL). An overnight 100 mL culture was diluted in 6 L of LB and grown at 37°C to an OD_600_ of 0.5–0.6. Subsequently, heterologous expression was induced with 0.5 mM IPTG (isopropyl β-D-1-thiogalactopyranoside) and the culture was transferred to 37°C for 4 h. Thereafter, cells were harvested at 3,800 g for 5 min at room temperature. The pellet was resuspended in buffer A (50 mM NaH_2_PO_4_, 300 mM NaCl, 30–80 mM imidazole) containing protease inhibitor (1 tablet in 2 mL *MilliQ*-purified water). Disruption of cells was carried out on ice by sonication and 30 μL of DNase I (10 mg/mL) and 30 μL of RNase (5 mg/mL) were added and incubated on ice for 20 min. Cell debris were removed by centrifugation at 48,000 g (JA-25.50 rotor) for 40 min at 4°C and the supernatant was collected. The purification was carried out with an Ä*kta Prime* System (*GE Healthcare*) with 5 mL of Ni-NTA according to the manufacturer's instructions. All steps were performed at 4°C. The column was charged with the supernatant and subsequently washed with 15 column volumes of buffer A. The attached proteins were eluted by ligand exchange employing a step gradient of 10, 20, 30, 50, and finally 100% of buffer B (50 mM NaH_2_PO_4_, 300 mM NaCl, 500 mM imidazole). Fractions containing specific NtcA were visualized by Coomassie-stained 12% SDS-PAGE.

#### Molecular size exclusion chromatography

The protein solution was concentrated to a volume of 5 mL by centrifugation with *Centriprep* filters (MWCO 10 or 30 kDa) (*Millipore*) at 1,500 g and 4°C. The concentrated sample was loaded on a *Sephadex75* size exclusion chromatography column for further purification. Pure protein fractions were further concentrated and stored in HBS-E buffer at 4°C not exceeding 4 weeks for SPR measurements, or in HBS-E with 50% (v/v) glycerol at −20°C for longer terms storage.

### Isothermal titration calorimetry

All dsDNA samples were prepared from 1 mM solutions for each oligonucleotide (*Integrated DNA Technologies*) including the wild-type or mutated binding site sequence for NtcA from the different *Prochlorococcus* strains. The specific DNA fragments contained the binding site for NtcA from the promoter region of *glnA* and the reference oligonucleotides contained this binding site with mutations (Table 1). Each pair of oligonucleotides was mixed in an equimolar solution and subjected to 5 min at 95°C, followed by a 3-h temperature gradient from 95 to 25°C, decreasing 0.5°C each step. 0.5 mM dsDNA stock solutions were obtained for each NtcA binding site sequence.

**Table 1:** Wild-type and mutated oligonucleotides corresponding to the promoter of the *glnA* gene in *Prochlorococcus* sp. strains MIT9313, SS120, and MED4.

**Oligonucleotide (5′->3′)**	**Function**	**Technique**
TAGAAGGTACCTGTTGCTACAAAAAG	Forward *glnA* promoter MIT9313 (wild-type)	ITC, SPR, EMSA
CTTTTTGTAGCAACAGGTACCTTCTA	Reverse *glnA* promoter MIT9313 (wild-type)	ITC, SPR, EMSA
TAGAAGAGAAAGGATCCGCAAAAAG	Forward *glnA* promoter MIT9313 (mutated)	ITC, SPR, EMSA
CTTTTTTGCGGATCCTTTCTCTTCTA	Reverse *glnA* promoter MIT9313 (mutated)	ITC, SPR, EMSA
CCAATAGTCACAAAAAGTACTTATTG	Forward *glnA* promoter SS120 (wild-type)	ITC, SPR, EMSA
CAATAAGTACTTTTTGTGACTATTGG	Reverse *glnA* promoter SS120 (wild-type)	ITC, SPR, EMSA
CCAATAAGCCCCACACGGCATTATTG	Forward *glnA* promoter SS120 (mutated)	ITC, SPR, EMSA
CAATAATGCCGTGTGGGGCTTATTGG	Reverse *glnA* promoter SS120 (mutated)	ITC, SPR, EMSA
AAAAAGTTACCTTTGATACATAAT	Forward *glnA* promoter MED4 (wild-type)	ITC
ATTATGTATCAAAGGTAACTTTTT	Reverse *glnA* promoter MED4 (wild-type)	ITC
AAAAACTAACCTTTGACTGATAAT	Forward *glnA* promoter MED4 (mutated)	ITC
ATTATCAGTCAAAGGTTAGTTTTT	Reverse *glnA* promoter MED4 (mutated)	ITC

*The DNA fragments are shown with the nucleotides subjected to mutations, listing the function for each DNA fragment and the technique where they have been used. Red: Binding site conserved (wild-type)/Blue: Binding site conserved mutated*.

The interaction between the different NtcA variants and dsDNA was characterized using an Auto-iTC200 microcalorimeter (MicroCal, Malvern). Titrations were performed at 25°C. Protein in the calorimetric cell at 5–10 μM in a monomer basis (5 μM for NtcA SS120 and NtcA MED4 experiments, 10 μM for MIT9313 experiments) was titrated with dsDNA at 50–100 μM (50 μM for NtcA SS120 and NtcA MED4 experiments, 100 μM for MIT9313 experiments). All solutions were degassed at 15°C for 2 min before each assay. A sequence of 2 μL-injections of titrant solution every 150 s was programmed and the stirring speed was set to 750 rpm. Different experiments increasing 2OG concentration (from 0 to 10 mM) in the calorimetric cell were performed. Each single experiment provided the apparent dissociation constant ${\text{K}}_{\text{d}}^{\text{app}}$ for NtcA interacting with dsDNA at a certain 2OG concentration (Supplementary Figure 2). Dissociation constants were estimated through non-linear least squares regression analysis of the experimental data employing a single ligand binding site model (1:1 dsDNA:dimer stoichiometry) implemented in Origin (OriginLab).

From each titration, the apparent dissociation constant for the NtcA-dsDNA interaction in the presence of 2OG, ${\text{K}}_{\text{d}}^{\text{app}}$, was determined. If the binding of dsDNA is affected by 2OG, the dissociation constant may increase (negative cooperativity) or decrease (positive cooperativity). Considering the ternary equilibrium between NtcA, dsDNA, and 2OG, the ${\text{K}}_{\text{d}}^{\text{app}}$ for the NtcA-dsDNA interaction will explicitly depend on the concentration of 2OG:

$$\begin{array}{l} K_{d}^{app}=K_{d}\frac{1+\frac{\left[oxo\right]}{K_{d,oxo}}}{1+\alpha \frac{\left[oxo\right]}{K_{d,oxo}}} \end{array}$$

where K_d_ is the (intrinsic) dissociation constant for the NtcA-dsDNA interaction in the absence of 2OG, [oxo] is the concentration of 2OG, K_d,oxo_ is the dissociation constant for the NtcA-2OG interaction, and α is the cooperativity interaction constant (α > 1 indicates positive cooperativity, whereas α < 1 indicates negative cooperativity) (Velazquez-Campoy et al., 2006). The limit value for the dissociation constant ${\text{K}}_{\text{d}}^{\text{app}}$ at very high 2OG concentration is K_d,eff_ = K_d_/α. The cooperative effect of both ligands is reciprocal, and, therefore, the dissociation constant for 2OG will also be affected by the presence of dsDNA. Thus, the limit value for the dissociation constant K_d,oxo_ at very large dsDNA concentration is equal to K_d,oxo_/α. This last value can be observed in the ${\text{K}}_{\text{d}}^{\text{app}}$ vs. [oxo] plot as the effective 2OG concentration, K_d,oxo, eff_, at which half-maximal change in ${\text{K}}_{\text{d}}^{\text{app}}$ is observed. Then, K_d,oxo, eff_ = K_d,oxo_/α is the effective K_d,oxo_, that is, the concentration of 2OG at which the cooperative effect (reduction of ${\text{K}}_{\text{d}}^{\text{app}}$ for dsDNA due to the presence of 2OG) is observed at half extent (or the dissociation constant for the NtcA/2OG interaction at very high dsDNA concentration). ${\text{K}}_{\text{d}}^{\text{app}}$ as a function of 2OG concentration can be fitted using the previous equation in order to estimate K_d_, K_d,oxo_, and α as adjustable parameters through non-linear least squares regression. The cooperative effect of 2OG and dsDNA is reciprocal: if 2OG increases the binding affinity of dsDNA, then, dsDNA increases the binding affinity of 2OG in the same extent.

### Surface plasmon resonance

All SPR measurements were carried out on a *BIACORE X* (*GE Healthcare*) instrument at 25°C. All 26 nt oligonucleotides were purchased from *MWG Eurofins/Ebersberg*. The forward strand was biotinylated at the 5′end. The DNA fragments used are summarized in Table 1.

Equal amounts of complementary strands (5 μM) were mixed in PCR tubes and hybridized in the heat block for 20 min at 80°C. Afterwards, the heat block was set at 60°C, then cooled down (ca. 20 min) and finally switched off. For the visualization of the hybridization, a 20% polyacrylamide gel was used.

#### SPR with sensor chip CM5 with neutroavidin

##### Surface preparation

The *Sensor Chip CM5* (*GE Healthcare*) was equilibrated at room temperature for 15–30 min in order to prevent condensation of water vapor on the chip surface. The *BIACORE* instrument was prepared with HBS-EP running buffer. The *Sensor Chip* was docked in the instrument. The next step was the coupling of the Neutroavidin in both flow cells (1 and 2). First, the chip was activated injecting 50 μL of EDC (1-ethyl-3-(3-dimethylaminopropyl)-carbodiimide) and 50 μL of NHS (N-hydroxysuccionimide). Then, 50 μL of 5 μM Neutroavidin (1 mg/mL) dissolved in 10 mM Na-Acetate was injected, followed by 35 μL of 1 M ethanolamin.

##### Sample injection

The samples containing NtcA from *Prochlorococcus* sp. SS120 and MIT9313 were diluted from the stock to 1 μM with HBS-EP buffer. To minimize sample dispersion at the beginning and the end of the injection, it is recommended to introduce a small volume of air with the sample (about 5 μL each) into the pipette tip before loading. This will separate the sample from running buffer and prevent mixing. The protein was injected with a flow rate of 5 μL/min. The interaction was analyzed with the program *Biacore X Control Software*, and the results evaluated using the software *BIAevaluation*.

To analyze the effect of 2OG on the binding between NtcA and the *glnA* promoter, 20 mL of 2OG were prepared in HBS-EP buffer as the concentration required, adjusting the pH to 7.4 with 5% of NaOH. The system was equilibrated with new buffer prior protein injection.

### Electrophoretic mobility shift assay (EMSA)

EMSA studies were carried out as described (Napolitano et al., 2012). Gels were stained with *SyberSafe* (*Invitrogen)* following the manufacturer instructions. The images were captured with a *ChemiDoc* system (*Bio-Rad)*.

### Genomic sequences and phylogenetic analysis

Selected cyanobacterial *ntcA* sequences were retrieved from CYORF (http://cyano.genome.jp/), Cyanobase (http://genome.microbedb.jp/cyanobase/), Integrated Microbial Genomes (https://img.jgi.doe.gov) and NCBI (http://www.ncbi.nlm.nih.gov/) databases. Protein BLAST analysis were performed using NCBI BLASTp tool (http://blast.ncbi.nlm.nih.gov/Blast.cgi?PAGE=Proteins). The software MEGA 7 (Kumar et al., 2016) was used to align the deduced protein sequences by using the MUSCLE method (using default settings) and a phylogenetic tree was obtained by using the Maximum Likelihood method. Further details are provided in the legend of **Figure 6**.

### Statistical analysis

The results are shown with error bars corresponding to the standard deviation. Significance of data was assessed by using the Student's *T*-test, and indicated in figures with asterisks: ^*^ means *p* ≤ 0.05; ^**^ means *p* ≤ 0.01.

## Results

2OG is the molecule responsible for the control of the C/N balance in cyanobacteria (Herrero et al., 2001; Muro-Pastor et al., 2001; Tanigawa et al., 2002; Vazquez-Bermudez et al., 2002; Flores and Herrero, 2005; Flores et al., 2005; Luque and Forchhammer, 2008). We have described previously its role in *Prochlorococcus* sp. PCC 9511, a strain genetically identical to MED4 (Domínguez-Martín et al., 2014). However, no specific study has been carried out thus far addressing the possible diversity of the role of 2OG within a cyanobacterial genus. Therefore, we decided to assess how this metabolite might impact the balance of C/N in different strains of *Prochlorococcus* under diverse conditions. To this goal, we studied first the azaserine effects on key enzymes and genes involved in the C/N metabolism in cyanobacteria; then proceeded to examine the possible diversity in the requirements of 2OG for the interaction between the NtcA transcriptional factor and the promoter of the *glnA* gene. Three model *Prochlorococcus* strains were selected for this work: MED4 (high light-adapted), SS120 and MIT9313 (both low light-adapted), with different evolutionary origins, as described above.

### Effect of azaserine on the metabolism of *Prochlorococcus* sp. SS120, MIT9313 and MED4

#### Effect of azaserine on the regulation of GS and ICDH

GS (EC 6.3.1.2) catalyzes the first step of the central pathway for ammonium assimilation in most organisms, including cyanobacteria; i.e., the ATP-dependent synthesis of glutamine from glutamate and ammonia. The enzyme isocitrate dehydrogenase (ICDH; EC 1.1.1.42) catalyzes the oxidative decarboxylation of isocitrate to produce 2OG. The possible variability between *Prochlorococcus* strains with respect to their regulatory mechanisms was assessed by studying the effect of different concentration of azaserine on these two key enzymes, GS and ICDH, using three different strains: MED4, MIT9313, and SS120.

**Figure 1:**
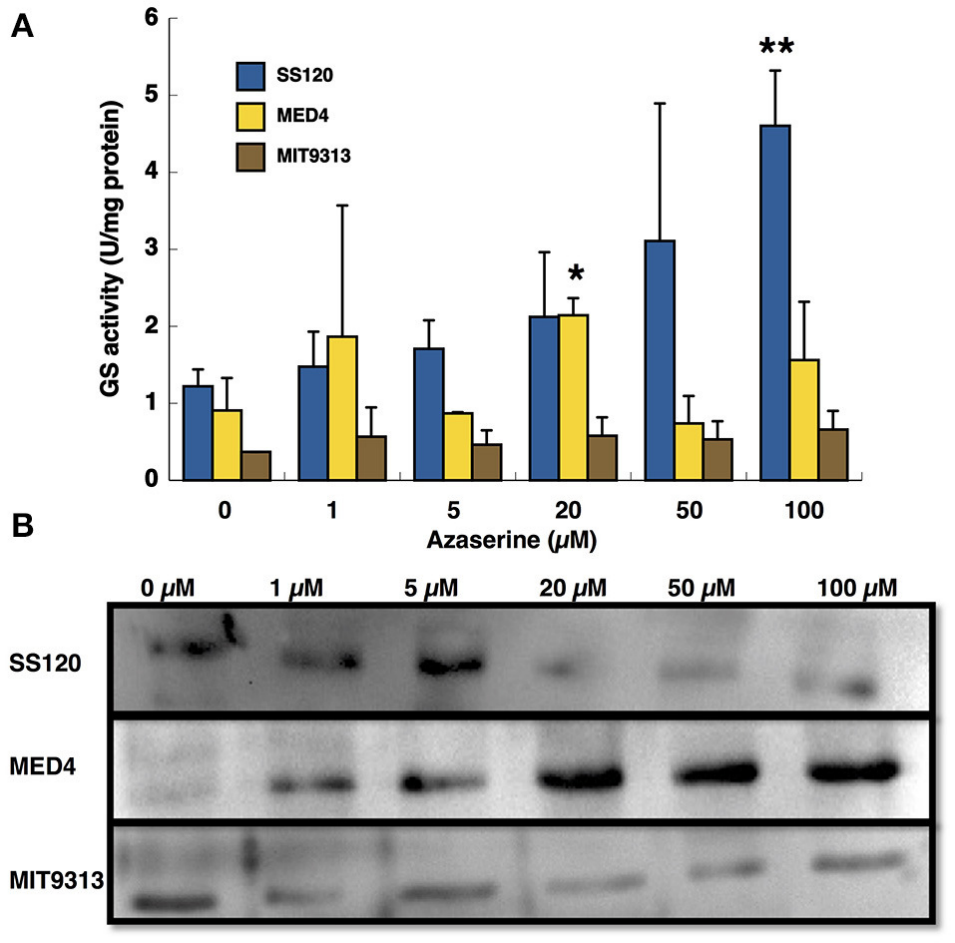
Effect of different concentration of azaserine on GS activity **(A)** and GS protein concentration **(B)** in different strains from *Prochlorococcus*. The different concentrations of azaserine were added to the cultures and cells were collected after 24 h of treatment. **(A)** is a representation of three independent biological replicates of each strain. Error bars correspond to standard deviation. **(B)** corresponds to a Western blotting using anti-GS antibodies.

Figure 1 shows that the response of GS transferase activity (Figure 1A) and the amount of GS (Figure 1B) to the different concentrations of azaserine is quite different depending on the strain. In the case of SS120, the activity increased with the concentration of azaserine (maximum level at 100 μM azaserine, *p* = 0.0018), while the other strains show a quite different pattern. In *Prochlorococcus* sp. MIT9313, the activity did not change with the concentration of azaserine. In MED4, GS activity only showed a significant increase after 20 μM azaserine addition.

GS concentration measured by Western blotting also showed three different patterns (Figure 1B): in MED4 the enzyme concentration increased with the concentration of the inhibitor but in contrast, GS from SS120 decreased. In MIT9313 the concentration of the enzyme showed little changes, in good agreement with the results observed for GS activity.

**Figure 2:**
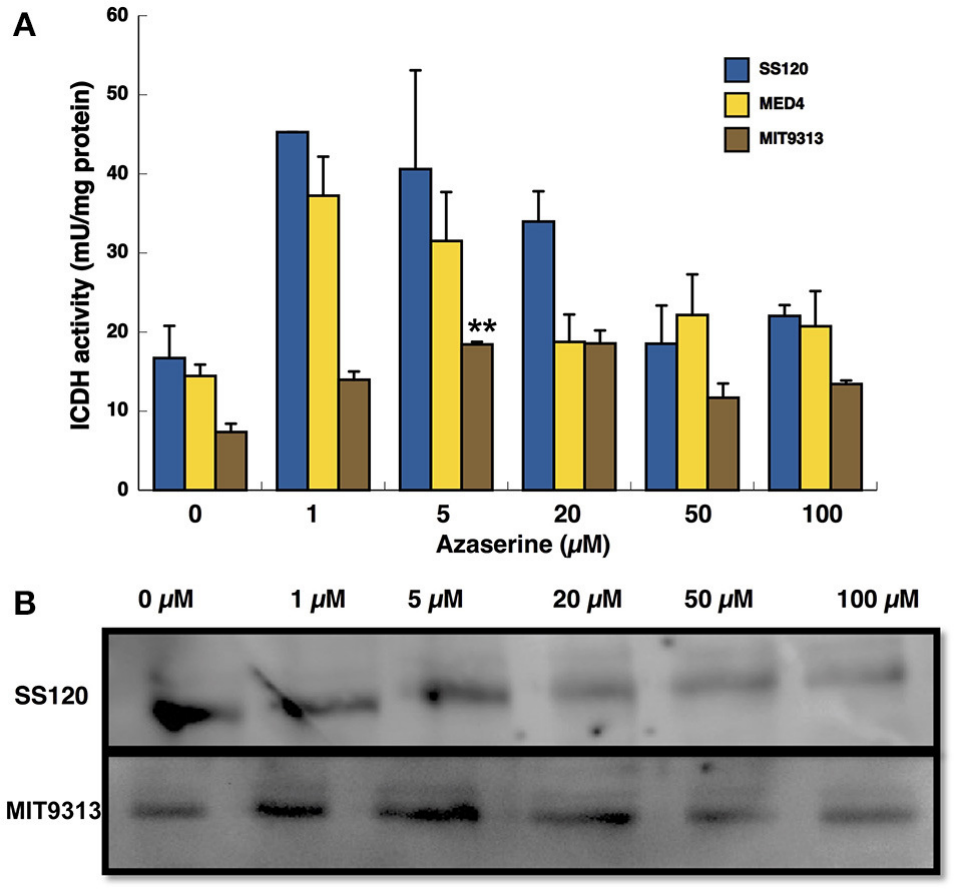
Effect of different concentration of azaserine on ICDH activity **(A)** and ICDH protein concentration **(B)** in different strains of *Prochlorococcus*. The different concentrations of azaserine were added to the cultures and cells were collected after 24 h of treatment. **(A)** is a representation of three independent biological replicates of each strain. Error bars correspond to standard deviation. **(B)** corresponds to a Western blotting using anti-ICDH antibodies.

ICDH activity was measured under the same conditions like GS (Figure 2A). The pattern was similar in the case of *Prochlorococcus* sp. SS120 and MED4, the activity being higher at low concentration of azaserine, although it was not reflected in the Western blotting for SS120 shown in Figure 2B. In the case of MIT9313, the activity increased significantly with azaserine concentration up to 5 μM (*p* = 0.0001; Figure 2A), and the same was observed for the concentration of the enzyme (Figure 2B).

The high amino acid sequence conservation of both GS and ICDH from the three *Prochlorococcus* strains studied here (Supplementary Figures 3, 4, respectively) suggests that the differences described above are not due to changes in structure or physico-chemicals properties of the enzymes, but to different regulatory mechanisms, probably derived from evolutionary pressures.

#### Effect of azaserine on *ntcA* expression

Since azaserine had a remarkable diversity of effects on enzyme regulation, we further studied this process by measuring the expression of the global nitrogen regulator, *ntcA* (encoding the transcriptional factor NtcA, which controls the expression of both *glnA* and *icd*, coding for glutamine synthetase and isocitrate dehydrogenase, respectively). We measured its expression by qRT-PCR in culture samples collected 24 h after the addition of different azaserine concentrations (0–100 μM).

**Figure 3:**
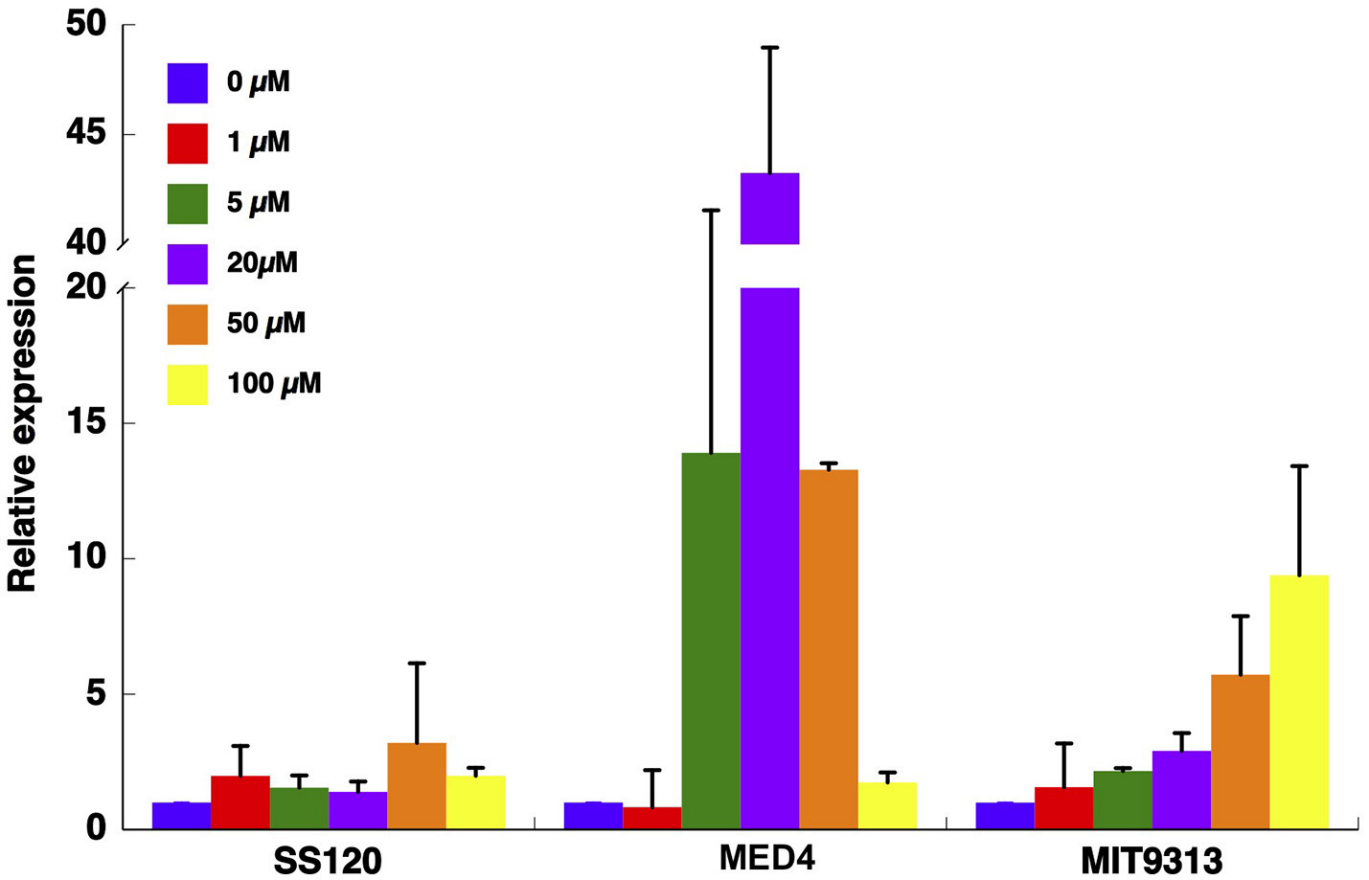
Effect of different concentration of azaserine on *ntcA* expression in different strains of *Prochlorococcus*. The different concentrations of azaserine were added to cultures. Samples were collected after 24 h, and gene expression was measured by qRT-PCR. Data are the average of four independent biological replicates. Error bars correspond to standard deviation.

The *ntcA* expression pattern was very different depending on the studied *Prochlorococcus* strain (Figure 3). While in SS120 the expression did not significantly change with the concentration of azaserine, in MED4, *ntcA* expression peaked at 20 μM azaserine (*p* = 0.0001), with significant increases also at 5 and 50 μM (*p* = 0.0279 and *p* = 0.0001, respectively). It is worth noting that the increase in *ntcA* expression observed in this strain was much higher than either in SS120 or MIT9313 (i.e., almost 5-fold higher than in MIT9313). In MIT9313 the expression of *ntcA* increased with the concentration of the inhibitor, being significantly higher at 50 and 100 μM (*p* = 0.0047 and *p* = 0.0059, respectively).

### Study of the interaction of NtcA with *glnA* promoter DNA in *Prochlorococcus* sp. MED4, SS120, and MIT9313

Since the enzymatic and gene expression results described above pointed out to differences in the regulation of the C/N balance between the studied *Prochlorococcus* strains, we decided to carry out a comparative study of the interaction between the global nitrogen regulator protein in cyanobacteria, NtcA, and one of the promoters subjected to its regulation, that of the *glnA* gene. To this goal, we performed the heterologous overexpression of *ntcA* from *Prochlorococcus* sp. MED4, SS120, and MIT9313. The overexpressed NtcA proteins were used to carry out isothermal titration calorimetry (ITC), surface plasmon resonance (SPR), and electrophoretic mobility assays (EMSA) studies, in order to determine the properties of the NtcA-*glnA* promoter interaction and their dependence on 2OG concentration in each *Prochlorococcus* strain.

#### Isothermal titration calorimetry studies

**Figure 4:**
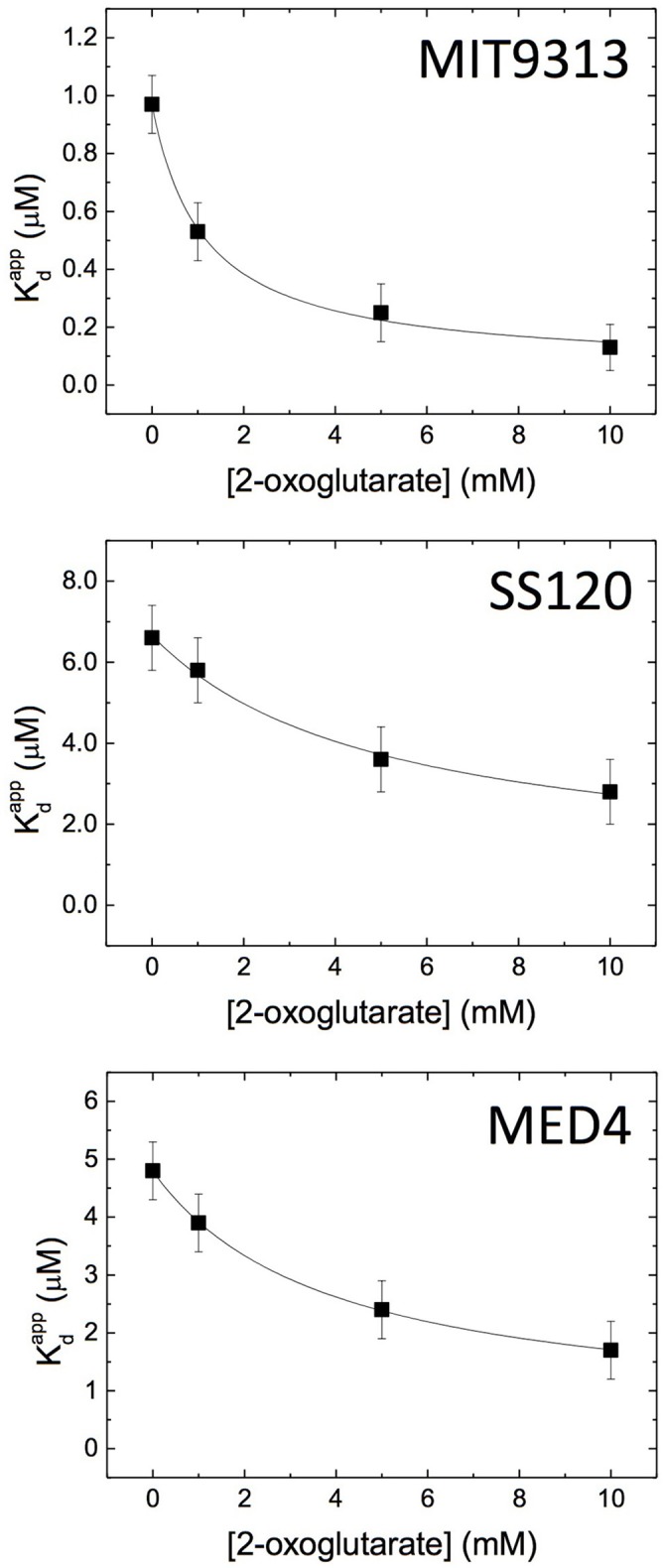
Isothermal titration calorimetry study of the NtcA-P*glnA* interaction in the *Prochlorococcus* MED4, MIT9313 and SS120 strains. Apparent dissociation constant (${\text{K}}_{\text{d}}^{\text{app}}$) of the interaction of NtcA with the wild-type *glnA* promoter DNA was determined in the presence of different concentrations of 2OG. Further details are shown in Table 2.

Wild-type and mutated versions of the promoter of *glnA* (to alter the canonical NtcA binding site) from *Prochlorococcus* sp. MED4, MIT9313, and SS120 were chemically generated so that the mutated promoters avoided the NtcA binding (Table 1). The dissociation constant K_d_ for the NtcA—*glnA* promoter interaction was determined in the three strains (Figure 4, Supplementary Figure 2), using four concentrations of 2OG (0, 1, 5, and 10 mM). We observed clear differences between MIT9313, SS120, and MED4 (Table 2, Figure 4): in the three strains, NtcA interacts with the *glnA* promoter in the absence of 2OG (Figure 4). The binding affinity of NtcA for the promoter in MIT9313 (K_d_ = 0.97 μM; the lower K_d_, the higher the binding affinity) is higher than in SS120 (K_d_ = 6.7 μM) and MED4 (K_d_ = 4.8 μM). Also in the three strains, NtcA interacts with 2OG. The binding affinity for NtcA/2OG (reflected by K_d,oxo_; the lower K_d,oxo_, the higher the binding affinity) is also higher in MIT9313 (Table 2). This means that the effect of 2OG on NtcA interacting with the *glnA* promoter will be observed at lower 2OG concentrations in MIT9313 than in SS120 and MED4.

**Table 2:** Isothermal titration calorimetry parameters of the NtcA—*glnA* promoter interaction in *Prochlorococcus* MIT9313, SS120, and MED4.

		**K_d_ (μM)**	**K_d,oxo_ (mM)**	**α**	**K_d,eff_ (μM)**	**K_d,oxo,eff_ (mM)**
MIT9313	DNA1	0.97 ± 0.09	19 ± 2	17 ± 2	0.06 ± 0.01	1.1 ± 0.2
	DNA2	2.1 ± 0.2	–	(1)	–	–
SS120	DNA3	6.7 ± 0.7	42 ± 3	8.4 ± 0.7	0.80 ± 0.2	5.0 ± 0.9
	DNA4	10 ± 2	–	(1)	–	–
MED4	DNA5	4.8 ± 0.4	36 ± 3	9.3 ± 0.8	0.5 ± 0.1	3.9 ± 0.7
	DNA6	9.5 ± 0.9	–	(1)	–	–

*Technical explanations for the meaning of the different constants are described in Materials and Methods. DNA1, DNA3, and DNA5 are DNA fragments including the glnA promoter for the MIT9313, SS120, and MED4 strains, respectively. DNA2, DNA4 and DNA6 are mutated versions of the same fragments where the NtcA binding site is missing. In the case of mutant dsDNA (DNA2, DNA4, and DNA6), K_d_ did not change with 2-oxoglutarate concentration, and therefore α ≈ 1 (no binding cooperativity between dsDNA and 2-oxoglutarate)*.

NtcA shows a positive cooperativity effect (α > 1; Table 2) between 2OG and the *glnA* promoter in all studied strains. NtcA from MIT9313 is more sensitive to 2OG, showing a higher binding affinity for 2OG (lower K_d,oxo_) and a larger cooperativity effect (higher α), resulting in a higher effective affinity to *glnA* promoter and 2OG in the presence of both ligands. In contrast, NtcA from both SS120 and MED4 behave similarly: they have lower binding affinity and lower cooperative effect from 2OG, compared to MIT9313. It is worth noting that the value of K_d_ for SS120 and MED4 is roughly 7- and 5-fold higher, respectively, than for MIT9313; these are significant changes, stressing the strong difference of the NtcA responsiveness to 2OG between these strains. For each NtcA variant, the binding affinity for the mutated promoter DNA is lower (higher K_d_) than that for the wild-type promoter DNA, and the cooperative effect from 2OG is absent (i.e., α ≈ 1, and increasing 2OG concentration hardly affects the binding affinity of mutated dsDNA; Table 2).

#### Surface plasmon resonance studies

The differences in the interaction of NtcA with *glnA* promoter DNA were also studied by Surface Plasmon Resonance, restricted in this case to the two low-light adapted *Prochlorococcus* strains (MIT9313 and SS120), since the behaviour of MED4 was very similar to SS120, as observed with ITC. As in the case of ITC, promoters of *glnA* from MIT9313 and SS120 were chemically generated (Table 1) and *ntcA* from *Prochlorococcus* sp. SS120 and MIT9313 were heterologously overexpressed (Supplementary Figure 1). The first step was the hybridization of the oligonucleotides to the *glnA* promoter DNA for each strain, as shown in Supplementary Figure 5. To confirm that 2OG does not bind directly to the chip, a control experiment using 2OG without the protein was performed, and no change in the SPR relative units was observed.

**Figure 5:**
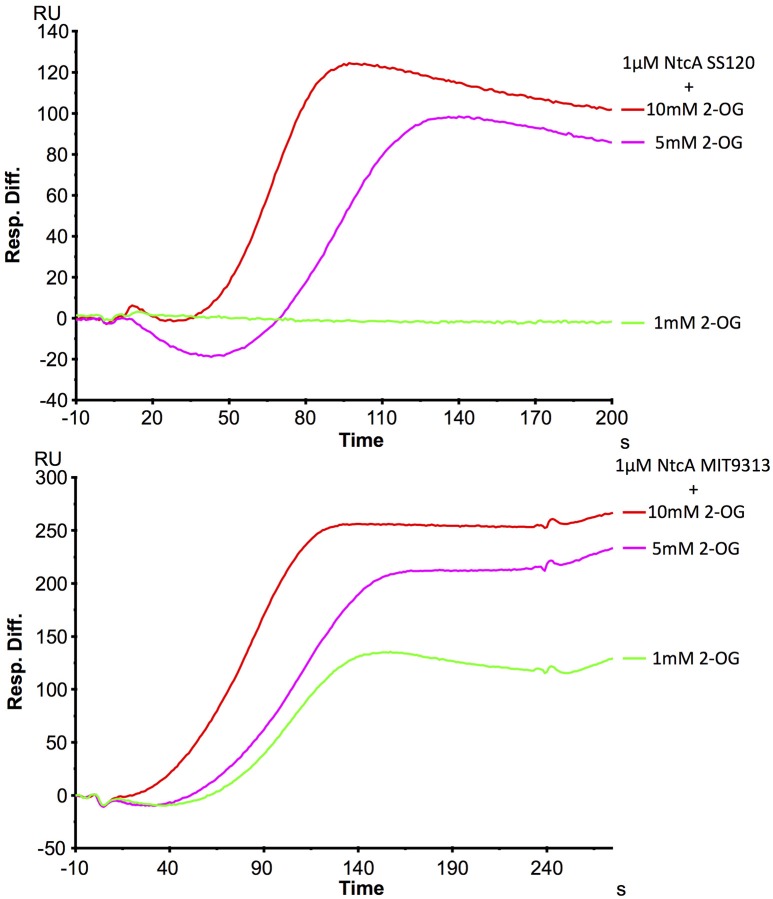
SPR analysis of the interaction between NtcA(His)_6_ and the promoter for *glnA* in *Prochlorococcus* sp. SS120 and MIT9313. This sensorgram shows the interaction of 1 μM of NtcA(His)_6_ with wild-type *glnA* promoter DNA and a rising concentration of 2OG. Relative units show the signal difference from FC2 (*glnA*) and an unspecific oligonucleotide coupled to the reference cell FC1. 1 μM NtcA was used in all assays, in the presence of 1 mM (green), 5 mM (pink), and 10 mM (red) 2OG.

The interaction NtcA-*glnA* promoter DNA was analyzed with two effector molecules, glutamine and 2OG. The results with different millimolar concentration of glutamine did not show any positive effect on the binding. On the contrary, 2OG had a positive effect on the binding, although it is clearly different between SS120 and MIT9313 (Figure 5). The binding appeared in MIT9313 with 1 mM 2OG and increased with the concentration of 2OG, however a higher level of 2OG was required for the binding in SS120. Although the shape of the sensorgrams hint to an influence of the charge of 2OG on the SPR measurements and consequently these results have to be interpreted with caution, they support the observations of our ITC studies, showing a lower responsiveness to 2OG in *Prochlorococcus* sp. SS120 strain with respect to MIT9313.

#### Electrophoretic mobility shift assays

In order to confirm the ITC and SPR results described above, we used EMSA under the same conditions and with the same purified proteins used in the SPR measurements.

**Figure 6:**
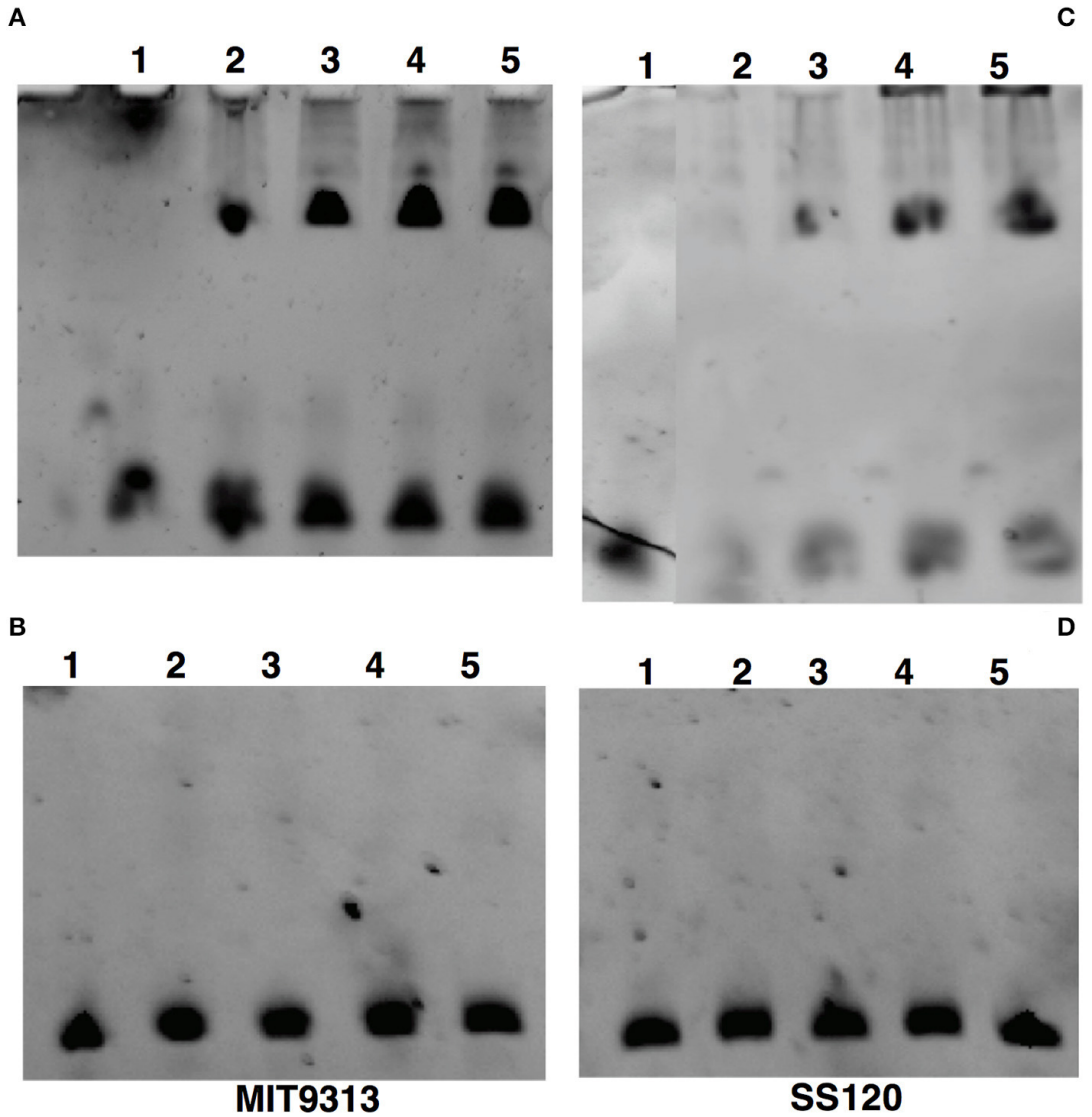
EMSA analysis of the specific binding of NtcA to the *glnA* promoter of *Prochlorococcus* sp. MIT9313 **(A,B)** and SS120 **(C,D)**. **(A,C)** The binding of 1 μM NtcA to the wild-type *glnA* promoter DNA was examined by EMSA analysis in the presence of the indicated concentrations of 2OG. **(B,D)** The binding of 1 μM NtcA in the presence of the indicated concentrations of 2OG to mutated *glnA* promoter. The samples had the following composition: Lane 1, *glnA*-promoter; lane 2, *glnA*-promoter + NtcA (1 μM); lane 3, *glnA*-promoter + NtcA (1 μM) + 2OG (1 mM); lane 4, *glnA*-promoter + NtcA (1 μM) + 2OG (5 mM); lane 5, *glnA*-promoter + NtcA (1 μM) + 2OG (10 mM).

Figure 6 shows that the EMSA results of the interaction were in good agreement to those obtained with ITC and SPR: the binding of NtcA from MIT9313 (Figure 6A) was enhanced with 2OG, being stronger when the concentration rises from 1 to 10 mM. In the case of NtcA from SS120, the binding needed a higher concentration of 2OG (Figure 6C). Figure 6B (MIT9313) and D (SS120) show the interaction between NtcA from both strains and the mutated *glnA* promoter DNA (Table 1). Our EMSA results showed no binding with the mutated promoter (Figures 6B,D), indicating that the GTAN_8/9_TAC sequence is specific for NtcA from *Prochlorococcus*. Nevertheless ITC studies, due to their higher sensitivy, showed a small level of binding between NtcA and the mutated promoter (Table 2).

### Phylogeny of NtcA in marine picocyanobacteria

**Figure 7:**
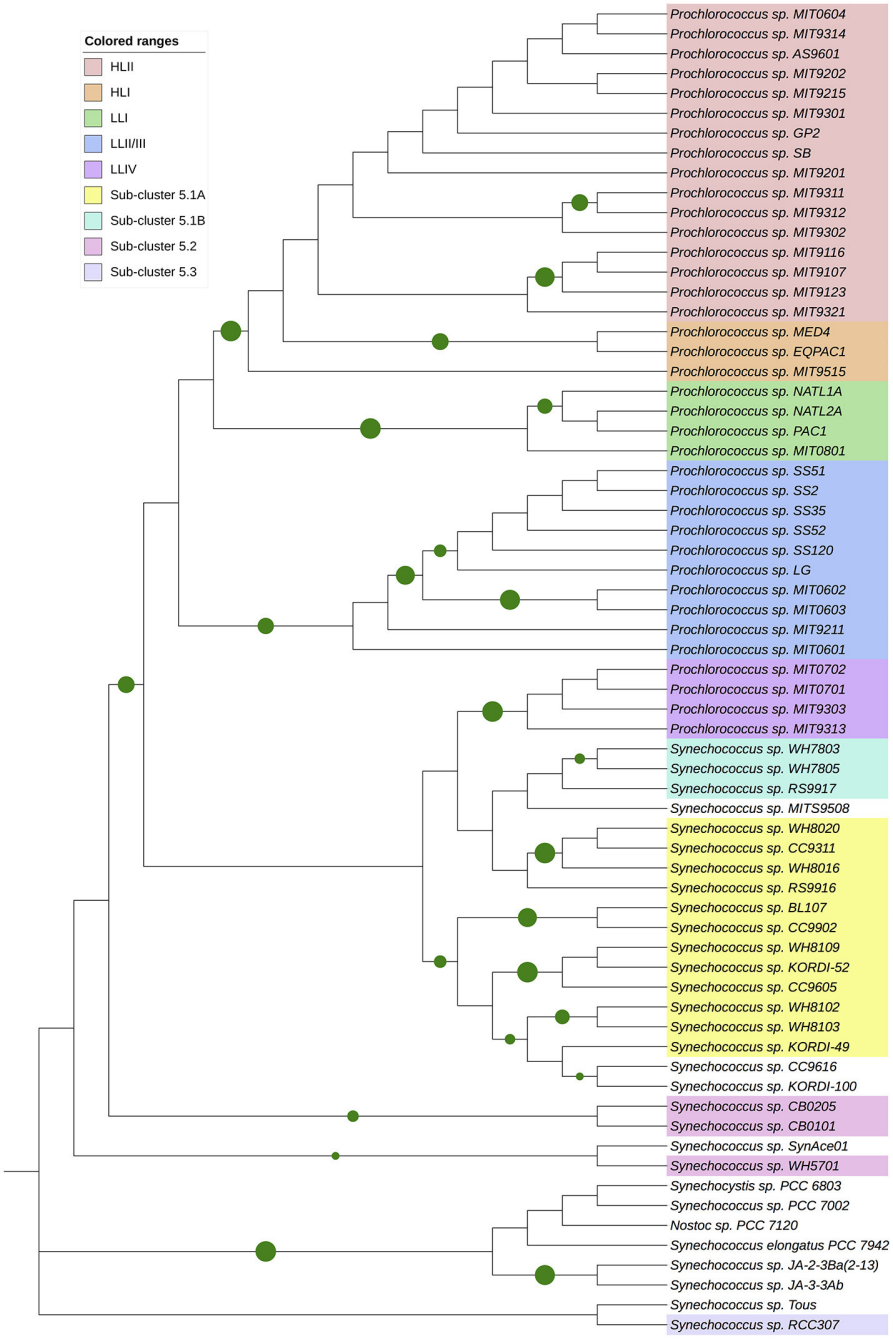
Phylogenetic tree based on cyanobacterial NtcA sequences. Molecular phylogenetic analysis by Maximum Likelihood method. The evolutionary history was inferred by using the Maximum Likelihood method based on the Whelan and Goldman model (Whelan and Goldman, 2001). The tree with the highest log likelihood (−3239.73) is shown. Initial tree(s) for the heuristic search were obtained automatically by applying Neighbor-Join and BioNJ algorithms to a matrix of pairwise distances estimated using a JTT model, and then selecting the topology with superior log likelihood value. Circle sizes correspond to bootstrap value (only values higher than 0.75 are shown). The analysis involved 67 amino acid sequences. All positions containing gaps and missing data were eliminated. There were a total of 210 positions in the final dataset. Evolutionary analyses were conducted in MEGA7 (Kumar et al., 2016), and the tree edited with iTOL (Letunic and Bork, 2016).

In order to study the evolution of the *ntcA* gene in the marine picocyanobacterial radiation, we retrieved a set of *ntcA* genes from marine *Synechococcus* and *Prochlorococcus* strains, together with sequences from freshwater model cyanobacterial strains, which were used as outgroup (i.e., *Synechocystis* sp. strain PCC 6803, *Synechococcus elongatus* strain PCC 7942, *Nostoc* sp. strain PCC 7120). The genes were translated to their amino acid sequence, aligned by using the MUSCLE method, and a phylogenetic tree was constructed with the MEGA 7 software, following the procedures described in the legend of Figure 7. The results are consistent with previous phylogenetic studies on *ntcA* (Penno et al., 2006), showing the progressive appearance of the different ecotypes in *Prochlorococcus* and *Synechococcus* (Figure 7).

Interestingly, the phylogeny of *ntcA* is in good agreement with previous models established for the evolution of the *Prochlorococcus* and *Synechococcus* genera (Moore et al., 1998; Rocap et al., 2002; Dufresne et al., 2008; Scanlan et al., 2009; Biller et al., 2015): both are closely related but show a clear separation.

A striking aspect of this phylogeny was the grouping of four *Prochlorococcus* strains (including MIT9313) of the LLIV clade with one of the subbranches corresponding to marine *Synechococcus* strains of the sub-clusters 5.1A and 5.1B, such as WH7803, highlighting the common origin of both genera and the earlier origin of MIT9313, which is considered as one of the oldest *Prochlorococcus* strains (Rocap et al., 2002). By contrast, the *ntcA* sequences of *Prochlorococcus* sp. strains MED4 and SS120 appear in different branches (Figure 7; Penno et al., 2006), corresponding to strains which diverged later in the *Prochlorococcus* phylogeny (Rocap et al., 2002; Penno et al., 2006).

## Discussion

The main goal of this work was to further study the differences in the regulatory response to the C/N balance between *Prochlorococcus* strains and to understand the molecular underpinnings of the process. 2OG is the molecule utilized by cyanobacteria to perceive the balance between C and N (Muro-Pastor et al., 2001). The binding affinity of the nitrogen control transcription factor, NtcA, is modulated by 2OG (Tanigawa et al., 2002; Vazquez-Bermudez et al., 2002). The role of 2OG as master regulator metabolite is supported by a large body of evidence in bacteria, archaea, and also eukaryotes (Huergo and Dixon, 2015). Therefore, we used several strategies to assess the effects of different 2OG concentration in the regulation of the C/N balance in three model strains of *Prochlorococcus*.

Early results from our team suggested that azaserine provoked differential changes depending on the *Prochlorococcus* strain (El Alaoui et al., 2001). The response of the three studied strains to increasing concentrations of azaserine was clearly different (Figures 1–3): MIT9313 showed little changes in the enzymatic activities, while SS120 responded with clear increases. By contrast, the expression of the gene encoding the global nitrogen regulator NtcA was increased in response to the 2OG concentrations in MIT9313, but was very little affected in SS120 (Figure 3). The strain MED4 showed an intermediate behavior. Overall, these results suggested that *Prochlorococcus* strains have different sensitivities to the increases in the 2OG concentration promoted by the azaserine addition.

NtcA interacts with the binding site GTA N_(8/9)_ TAC (Tolonen et al., 2006) in the *glnA* promoter DNA from *Prochlorococcus*. Our results showed that 2OG enhances the interaction between the transcription factor and DNA (Figures 4–6, Table 2), in agreement with the results described in other cyanobacteria (Luque et al., 1994; Tanigawa et al., 2002; Vazquez-Bermudez et al., 2002; Espinosa et al., 2006; Valladares et al., 2008; Kuniyoshi et al., 2011; Forcada-Nadal et al., 2014). Previous studies have addressed this kind of interaction, usually in a single freshwater model strain, such as *S. elongatus* PCC 7942 (Tanigawa et al., 2002; Vazquez-Bermudez et al., 2002), *Synechocystis* sp. PCC 6803 (Muro-Pastor et al., 2001; Forcada-Nadal et al., 2014), *Nostoc* sp. PCC 7120 (Laurent et al., 2005; Valladares et al., 2008; Zhao et al., 2010), or *Microcystis aeruginosa* PCC 7806 (Kuniyoshi et al., 2011). The modulation of the binding activity of nitrogen transcriptional regulators has also been shown in other bacterial groups (Beckers et al., 2005; Hasselt et al., 2011). However, our results showed that the sensitivity of NtcA to the 2OG binding differs in MIT9313 vs SS120 and MED4. Interestingly, by using EMSA and SPR, we could detect almost no binding at 1 mM 2OG in the case of the SS120 strain, while in MIT9313 the level of binding was already very significant (Figures 5, 6). When this interaction was monitored by ITC (Figure 4, Table 2), we observed a significantly lower equilibrium dissociation constant for the binding NtcA–*glnA* promoter DNA in MIT9313 than in both SS120 and MED4. Furthermore, the cooperativity effect of 2OG with NtcA in the binding (α in Table 2) was rougly 2-fold higher in MIT9313 than in SS120 or MED4. Besides, the K_d,oxo,eff_ constant (concentration of 2OG at which the cooperative effect is observed at half extent) shows that the requirements of 2OG for binding are also significantly lower in MIT9313 than in SS120/MED4.

These results evidence an important level of diversity in a critical process of transcriptional regulation, as the NtcA control in cyanobacteria. To our knowledge, such a diverse response in members of the same cyanobacterial genus has not been reported before, and suggests that even central mechanisms of regulation (as the system controlling the C/N balance of cyanobacteria, involving the proteins NtcA-P_II_-PipX) are subjected to fine tuning by evolutionary pressure.

Previous studies described the K_d_ constant (in the absence of 2OG) for the NtcA-*glnA* promoter binding in freshwater cyanobacterial strains, by using either EMSA (Reyes et al., 1997; Vazquez-Bermudez et al., 2002) or SPR (Forcada-Nadal et al., 2014). The reported K_d_ values were 0.052 μM in *S. elongatus* PCC 7942 (Vazquez-Bermudez et al., 2002), 0.025 μM (Reyes et al., 1997), and 0.057 μM (Forcada-Nadal et al., 2014) in *Synechocystis* PCC 6803 (compared to 0.97, 6.7, and 4.8 μM for *Prochlorococcus* sp. MIT9313, SS120 and MED4, respectively; Table 2). This means that both *S. elongatus* PCC 7942 and *Synechocystis* sp. PCC 6803 are more sensitive to 2OG than the three *Prochlorococcus* strains here studied, and it fits nicely in our hypothesis of progressive loss of responsiveness to 2OG.

In this view, we suggest that transcriptional regulation carried out by NtcA in a 2OG-dependent manner appeared early in the cyanobacterial radiation, since the branch leading to *Synechococcus elongatus* strain PCC 7942 diverged at the end of the Paleoproterozoic period (ca. 1750 millions of years ago), according to recent phylogenetic studies (Sanchez-Baracaldo et al., 2014). *Synechocystis* sp. strain PCC 6803 diverged later (in the Neoproterozoic era, ca. 750 million years ago; Sanchez-Baracaldo et al., 2014). Since this strain lives in changing freshwater habitats, it has conserved a similar 2OG-dependent NtcA system (with roughly the same K_d_ observed in PCC 7942).

The marine picocyanobacterial radiation occurred later in the evolution, giving place to the large diversity observed in the *Synechococcus* and *Prochlorococcus* genera (Scanlan et al., 2009). During that radiation, picocyanobacterial strains had to get adapted to different kinds of marine environments, from coastal—subjected to changing conditions—to rather stable oligotrophic gyres of the oceans. Presumably, the more diverse *Synechococcus* genera would include a range of regulatory C/N systems mediated by NtcA: from ecotypes adapted to variable habitats (where the NtcA system maintains a 2OG responsiveness similar to that of freshwater cyanobacterial strains), to oligotrophic habitats (where the NtcA system is gradually losing responsiveness to 2OG, as the *Synechococcus* ecotypes are more and more adapted to stable conditions). Future NtcA interaction studies in *Synechococcus* might allow to verify if this hypothesis holds true.

The whole *Prochlorococcus* genus appeared quite recently, in evolutive terms, and rapidly evolved a number of genetic features to adapt to oligotrophic, stable ocean environments. This is consistent with the phylogeny of *ntcA* (Figure 7; Penno et al., 2006), where the NtcA sequence of *Prochlorococcus* sp. MIT9313 sits next to early branching freshwater strains, while NtcA sequences from both MED4 and SS120 are located in late-branching positions of recently evolved strains.

In this context, we propose that one of the molecular adaptations in *Prochlorococcus* was to decrease the NtcA responsiveness to 2OG (Figures 4–6, Table 2), compared to the freshwater cyanobacterial ancestors. Specifically, the strain MIT9313 (corresponding to one of the earliest branching *Prochlorococcus* ecotypes, LLIV) diverged earlier than strain SS120 (ecotype LLII), while MED4 (ecotype HLI) is considered as one of the most recently evolved *Prochlorococcus* strains (Biller et al., 2015). The progressive loss of NtcA responsiveness to 2OG fits with this evolutive model, leading to a similar response in two rather different (SS120 is adapted to live at depth, while MED4 thrives near the ocean surface) but modern strains. The difference of habitats is reflected in the clearly different genotypes between these model *Prochlorococcus* strains (Dufresne et al., 2003; Rocap et al., 2003). The recent evolution of both SS120 and MED4 is illustrated by their similar, decreased responsiveness of their NtcA proteins to 2OG, when compared to the older MIT9313. This is consistent with previous work suggesting that the eMIT9313 clade has more complexity in their ability to respond to various stimuli (Kettler et al., 2007).

It is however difficult to establish a direct relationship between our observations in enzymes regulation (Figures 1, 2) and *ntcA* expression (Figure 3), with the results obtained in the NtcA interaction studies in the three *Prochlorococcus* strains (Figures 4–6). While an important level of diversity was detected in all those experiments, we found more changes in GS and ICDH in *Prochlorococcus* SS120 and MED4 than in MIT9313 (Figures 1, 2). In the experiments addressing *ntcA* expression, MED4 showed the most remarkable changes (with a peak at intermediate azaserine concentrations), while MIT9313 had a progressive increase with the maximum expression at the highest tested azaserine concentration (Figure 3).

This might be partially explained by the involvement of several key regulatory proteins in the C/N balance regulation of *Prochlorococcus*, as P_II_ (Palinska et al., 2002) and PipX (Espinosa et al., 2014), which have not been studied here and might contribute significantly to our observations. Another important factor might be the relatively short times of our studies (with samples taken 24 h after the beginning of the experiment), which might not be fully representative of the transcriptomic changes relevant in the context of a slow growing organism as *Prochlorococcus*, which divides once per day in the ocean (Vaulot et al., 1995). A global transcriptomic study addressing the effects of different N sources in *Prochlorococcus* (Tolonen et al., 2006) showed that the strain MED4 transcriptional response to N deprivation was rapid and transient, whereas the MIT9313 response was slower and sustained. This is coherent with our observations: given that the growth of *Prochlorococcus* is quite slow, a hypothesis is that it might take a long time to reach a 2OG concentration triggering the NtcA response to N deprivation, explaining the slow but continued transcriptional reply in MIT9313. The rapid but transient response to N limitation in MED4 might be mediated by other regulatory proteins as well (for instance, the high increase in *glnB* expression, which was not observed in MIT9313; Tolonen et al., 2006). Our team has studied the effect of nitrogen limitation in *Prochlorococcus* sp. strain SS120 by redox proteomics (Mcdonagh et al., 2012) and quantitative proteomics (Domínguez-Martín et al., 2017); future studies on the two other *Prochlorococcus* main model strains (MED4 and MIT9313) will allow to elucidate whether the transcriptional interstrain differences are also translated to the proteome.

The results here reported, together with different studies on the regulation of N metabolism in *Prochlorococcus* (El Alaoui et al., 2001, 2003; Gómez-Baena et al., 2001, 2015; Lindell et al., 2002; Tolonen et al., 2006; López-Lozano et al., 2009; Rangel et al., 2009; Gilbert and Fagan, 2010; Mcdonagh et al., 2012; Domínguez-Martín et al., 2014, 2017), suggest that the regulatory simplification observed in this cyanobacterium (Rocap et al., 2003; García-Fernández and Diez, 2004; García-Fernández et al., 2004; Steglich et al., 2008) is a complex process, involving the loss of regulatory proteins (Kettler et al., 2007) and the modulation in their sensitivity to key metabolites, such as exemplified here: reducing the NtcA responsiveness to 2OG means that *Prochlorococcus* triggers the N-stress response upon a higher threshold of 2OG (compared to other freshwater cyanobacterial strains), thus saving energy required for production of the N-stress induced proteins. These adaptive changes would mean lower energetic costs involved in the production and maintenance of the sensing and regulatory mechanisms, which would ultimately improve the fitness of *Prochlorococcus* in very oligotrophic environments.

A recent study proposed a decrease in nutrient flux density during the evolution of *Prochlorococcus* (Braakman et al., 2017), suggested by a decrease in its maximal growth rate and an increased efficiency in its use of limiting nutrients. Our results are consistent with this later feature: besides other strategies to increase efficiency in the use of low N concentrations (such as reducing the size of the genome and the guanine-cytosine content, Dufresne et al., 2005), the decreased use of amino acids with side chains rich in N (Grzymski and Dussaq, 2011), or the loss of genes required to assimilate oxidized forms of N (López-Lozano et al., 2002; Moore et al., 2002; Rocap et al., 2003), evolving a regulatory NtcA system less responsive to 2OG would optimize the utilization of N in oligotrophic oceans where conditions are rather stable.

## Concluding remarks

The results described in this paper show that a key aspect underlying the diversity of regulation of the C/N balance in *Prochlorococcus* is the decrease in responsiveness of NtcA to 2OG observed in recently evolved strains. However, besides NtcA, there are other regulatory cyanobacterial proteins involved in the control of N and C, such as the P_II_ protein (Forchhammer and Tandeau De Marsac, 1994), or PipX, which is another global regulator in cyanobacteria (Espinosa et al., 2014). Further studies should address the role of those additional players in the diversity of reponses from *Prochlorococcus*.

## Author contributions

MAD-M, AL-L, RC-G, AV-C, and GS performed research; MAD-M, AL-L, RC-G, AV-C, GS, AB, JD, and JG-F designed research, analyzed data, wrote, and approved the final version of the manuscript.

### Conflict of interest statement

The authors declare that the research was conducted in the absence of any commercial or financial relationships that could be construed as a potential conflict of interest.

## Supplementary material

Supplementary Figure 1 ITC titration of NtcA with dsDNA promoter of the *glnA* gene in the *Prochlorococcus* sp. strains MIT9313, SS120 and MED4. Calorimetric titrations for NtcA interacting with dsDNA sequences corresponding to the *glnA* promoter from the wild strains **(left)** MIT9313, **(middle)** SS120 and **(right)** MED4, in the absence of 2-oxoglutarate. Calorimetric thermograms (thermal power as a function of time) are shown in the upper plots, and binding isotherms (ligand normalized heat effects as a function of the molar ratio) are shown in the lower plots.

Supplementary Figure 2 Heterologous NtcA(His)_6_ purified from *Prochlorococcus* sp. SS120 and MIT9313. 1 and 3 μg of the purified protein were analyzed on a 12% polyacrylamide gel-SDS and stained with Coomassie solution.

Supplementary Figure 3 Multiple sequence alignment of GS from *Prochlorococcus* MIT9313, SS120 and MED4. The sequences in FASTA format were taken from CYORF and ClustalW2 from *EMBL-EBI* was used to make the alignment. (∗) Indicates positions which have a single, fully conserved residue; (:) indicates conservation between groups of strongly similar properties and (.) indicates conservation between groups of weakly similar properties. Highlighted amino acids residues involved in the biosynthetic reaction.

Supplementary Figure 4 Multiple sequence alignment of ICDH from *Prochlorococcus* MIT9313, SS120 and MED4. The sequences in FASTA format were taken from CYORF and ClustalW2 from *EMBL-EBI* was used to make the alignment. (∗) Indicates positions which have a single, fully conserved residue; (:) indicates conservation between groups of strongly similar properties and (.) indicates conservation between groups of weakly similar properties.

Supplementary Figure 5 Hybridization of *glnA* promoter. The 26 nt long oligonucleotides were analyzed on a 20% polyacrylamide gel. As control besides the *Peqlab ultra low range DNA standard* (0.01-0.3 kb) in the lane named MW, the single strand Forward (F) and Reverse (R) were loaded. The successfully hybridized samples are shown in lanes named 1, 2, and 3.

Supplementary Table 1 Oligonucleotides used for cloning and qRT-PCR.

## Abbreviations

EMSA: electrophoretic mobility shift assay
ITC: isothermal titration calorimetry
SPR: surface plasmon resonance
2OG: 2-oxoglutarate.
