# Supplementary figures and images for: Differential NtcA Responsiveness to 2-Oxoglutarate Underlies the Diversity of C/N Balance Regulation in Prochlorococcus
Source: Front Microbiol. 2018 Jan 9;8:2641. doi: 10.3389/fmicb.2017.02641 (PMC5767323; doi:10.3389/fmicb.2017.02641)

**SS120**

**MIT9313**

**kDa**

**50**

**40**

**30**

**25**

**20**

**15**

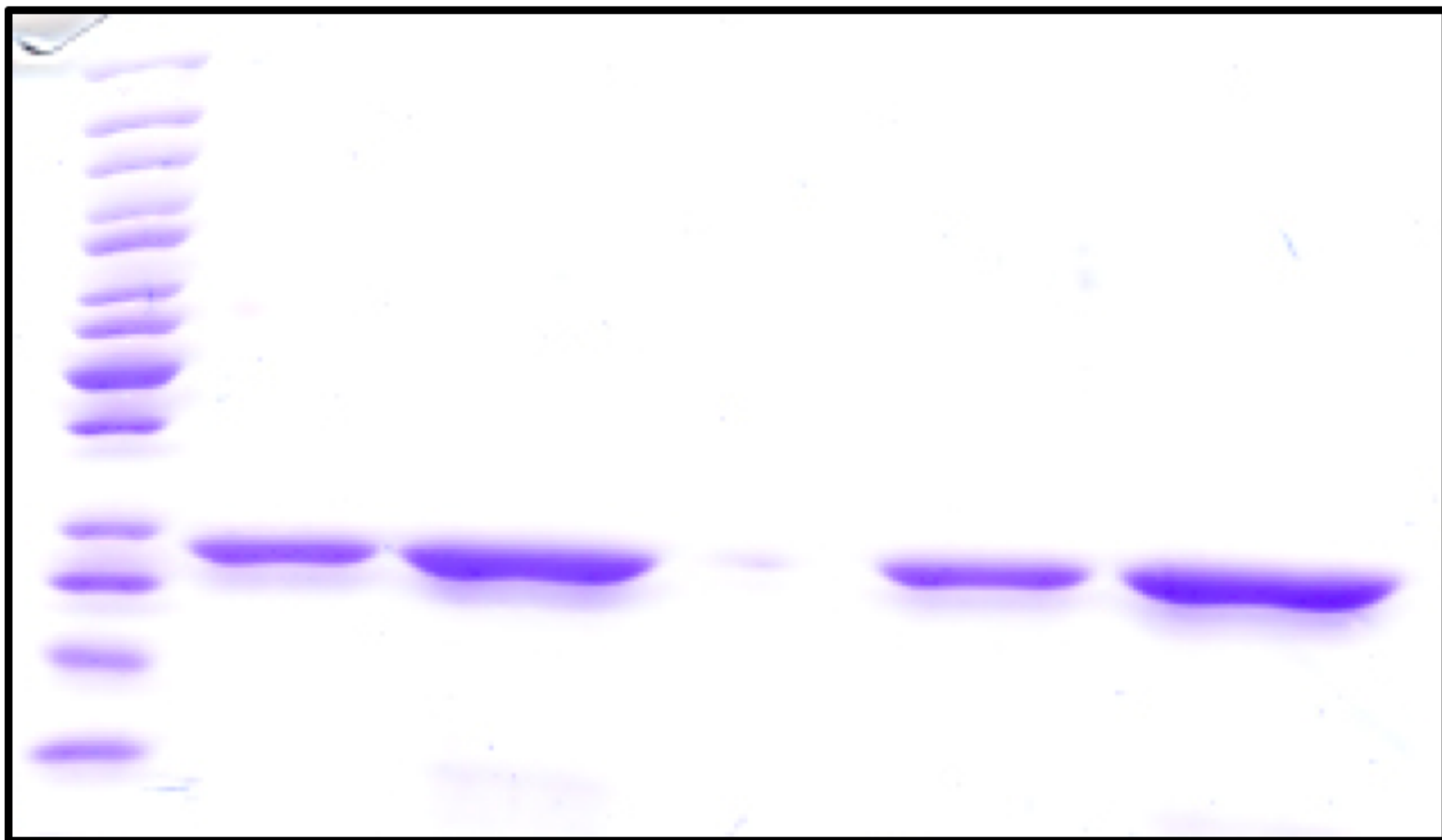

Supplement: Supplementary Figure 1 — ITC titration of NtcA with dsDNA promoter of the glnA gene in the Prochlorococcus sp. strains MIT9313, SS120 and MED4. Calorimetric titrations for NtcA interacting with dsDNA sequences corresponding to the glnA promoter from the wild strains (left) MIT9313, (middle) SS120 and (right) MED4, in the absence of 2-oxoglutarate. Calorimetric thermograms (thermal power as a function of time) are shown in the upper plots, and binding isotherms (ligand normalized heat effects as a function of the molar ratio) are shown in the lower plots. [file Image1.PDF]

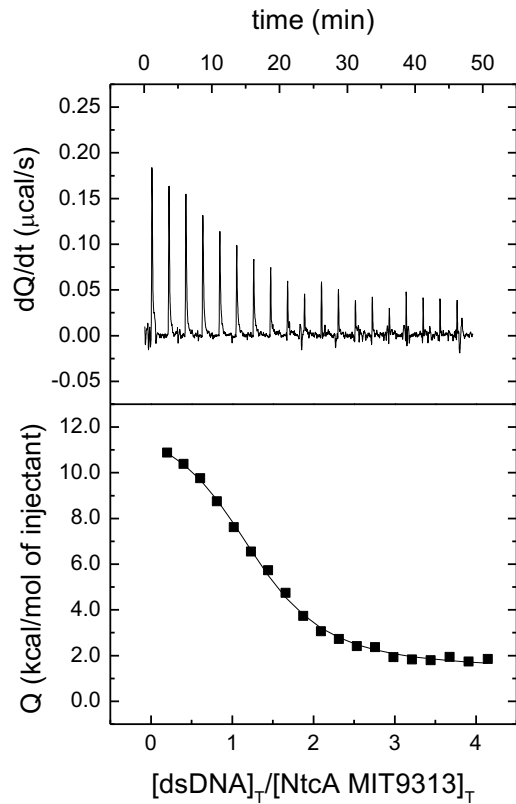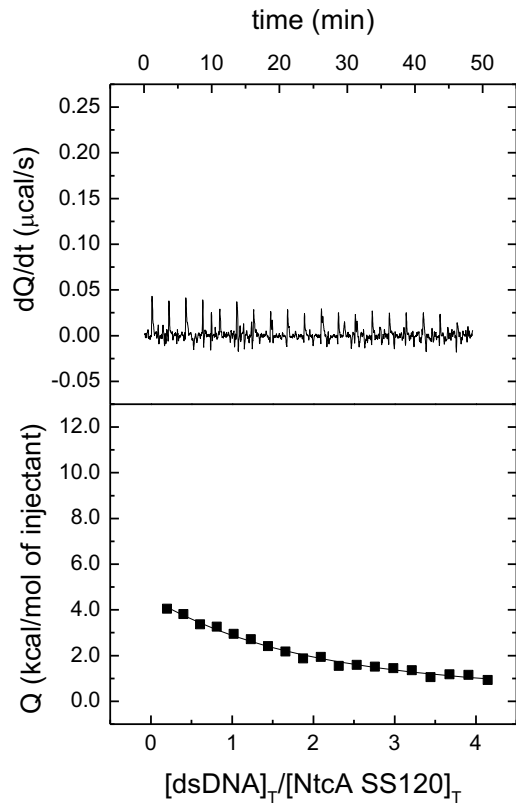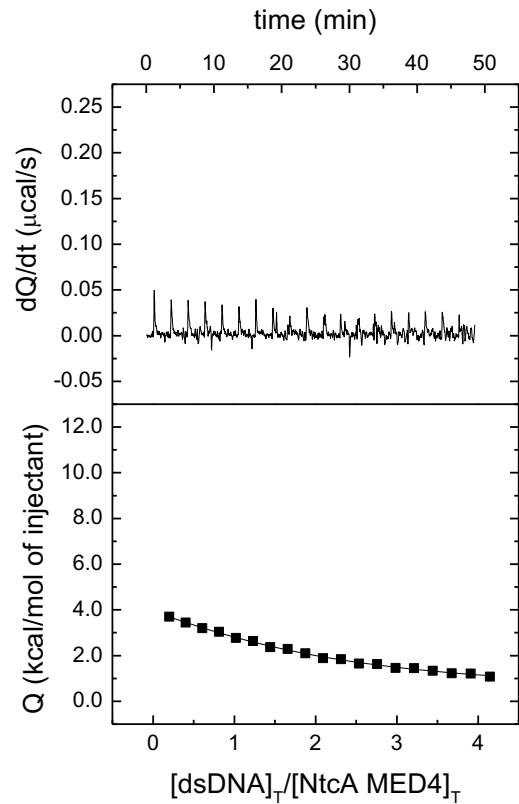

Supplement: Supplementary Figure 2 — Heterologous NtcA(His)6 purified from Prochlorococcus sp. SS120 and MIT9313. 1 and 3 μg of the purified protein were analyzed on a 12% polyacrylamide gel-SDS and stained with Coomassie solution. [file Image2.PDF]

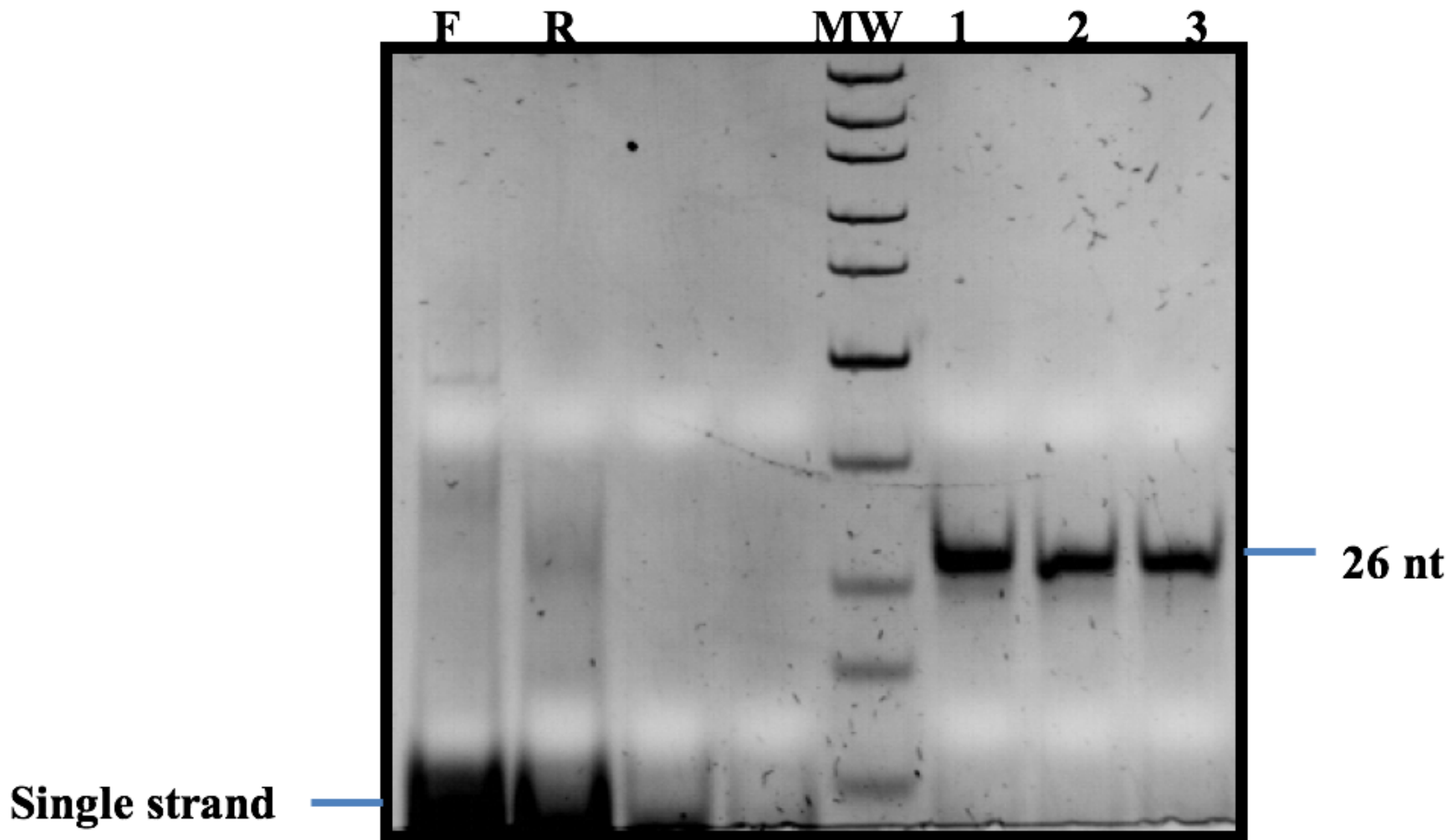

Supplement: Supplementary Figure 5 — Hybridization of glnA promoter. The 26 nt long oligonucleotides were analyzed on a 20% polyacrylamide gel. As control besides the Peqlab ultra low range DNA standard (0.01-0.3 kb) in the lane named MW, the single strand Forward (F) and Reverse (R) were loaded. The successfully hybridized samples are shown in lanes named 1, 2, and 3. [file Image5.PDF]
