# Supplementary material for: Differential NtcA Responsiveness to 2-Oxoglutarate Underlies the Diversity of C/N Balance Regulation in Prochlorococcus
Source: Front Microbiol. 2018 Jan 9;8:2641. doi: 10.3389/fmicb.2017.02641 (PMC5767323; doi:10.3389/fmicb.2017.02641)
Supplement: Supplementary Figure 3 — Multiple sequence alignment of GS from Prochlorococcus MIT9313, SS120 and MED4. The sequences in FASTA format were taken from CYORF and ClustalW2 from EMBL-EBI was used to make the alignment. (∗) Indicates positions which have a single, fully conserved residue; (:) indicates conservation between groups of strongly similar properties and (.) indicates conservation between groups of weakly similar properties. Highlighted amino acids residues involved in the biosynthetic reaction. [file Image3.PDF]

```
MED4      MAKSPQDVLSQIKDEGIELIDLKFTDIHGKWQHLTLTSDMIEEESFTEGLAFDGSSIRGW 60
SS120     MGKSPQDVLRQIKDEGIELIDLKFTDIHGKWQHLTVTSDMVDENSFKEGLAFDGSSIRGW 60
MIT9313   MGKSPQDVLRQIKDEGIELIDLKFTDLHGKWQHLTVCSDLIDEEAFANGLAFDGSSIRGW 60
          *.******* ****************:********: **:::*::* :************

MED4      KAINASDMSMVPDSSTAWIDPFYKHKTLSMICSIQEPRSGEPYNRCPRSLAQKALKYLDS 120
SS120     KAINESDMSMVPDSSTAWVDPFYRHKTLSLICSIQEPRSGEAYSRCPRSLAQKALAYLSN 120
MIT9313   KAINESDMDMVPDASTAWIDPFYRHKTLSLICSIREPRSGEPYARCPRALAQKALGYLAG 120
          **** ***.****:****:****:*****:****:******.* ****:****** ** .

MED4      TGIADTAFFGPEPEFFLFDDVRYDSKEGSCFYSVDTIEAPWNTGRTEEGGNLGYKIQYKE 180
SS120     TGLADSAYFGPEPEFFIFDDVRYDSKEGTSFYSVDTIEAPWNTGRAEEGGNLAYKIQYKE 180
MIT9313   TGLADTAFFGPEPEFFIFDDVRYNSGEGGCFYSVDTIEAPWNSGRIEEGGNLAYKIQLKE 180
          **:**:*:********:******:* ** .************:** ******.**** **

MED4      GYFPVAPNDTAQDIRSEMLLQMAELGIPTEKHHHEVAGAGQHELGIKFDSLISSADSVMT 240
SS120     GYFPVSPNDTAQDLRSEMLLLIGELGIPIEKHHHEVAGPGQHELGMKFASLISAADNVMT 240
MIT9313   GYFPVPPNDTAQDIRSEMLLLMGQLGIPMEKHHHEVAGAGQHELGMKFAELIEAADNVMI 240
          *****.*******:****** :.:**** *********.******:** .**.:**.**

MED4      YKYVVRNVAKKYGKTATFMPKPVFNDNGTGMHVHQSLWESGQPLFYGEGSYANLSQTARW 300
SS120     YKYVVRNIAKKYGKTATFMPKPVWNDNGTGMHVHQSLWKDGQPMFYGEGTYANLSQTAKW 300
MIT9313   YKYIVRNVARKYGKTATFMPKPVFNDNGTGMHVHQSLFKGGQPLFFGEGTYANLSQTARW 300
          ***:***:*:*************:*************::.***:*:***:********:*

MED4      YIGGILKHAPSFLAFTNPTTNSYKRLIPGFEAPVNLVYSEGNRSAAVRIPLTGPNPKAKR 360
SS120     YIGGILKHAPSFLAFTNPTTNSYKRLVPGFEAPVNLVYSQGNRSAAVRIPLTGPSPKAKR 360
MIT9313   YIGGILKHAPSFLAFTNPTTNSYKRLVPGFEAPVNLVYSQGNRSAAVRIPLTGPNPKAKR 360
          **************************:************:**************.*****

MED4      LEFRSGDALANPYLAFSVMMLAGIDGIKNQIDPGDGVDVDLFELPAEELSKIDTVPSSLN 420
SS120     LEFRSGDALANPYLAFSAMMMAGIDGIKNQIDPGDGVDVDLFELPEDELSKIATVPSSLN 420
MIT9313   LEFRPGDALANPYLAFSAMMMAGVDGIKNQIDPGDGFDEDLFELPEERLASIPTVPASLN 420
          ****.************.**:**:************.* ****** :.*:.* ***:***

MED4      DSLNALKADKDYLLAGGVFTEDFIDNFIDMKYEEVQQLRQRPHPHEFFMYYDA 473
SS120     NALEALKADNDYLTAGGVFDHDFINNFIEMKYEEVQQLRQRPHPHEFFMYYDA 473
MIT9313   GALEALNADKNYLMEGGVFTEDFIDNWIDIKYEEVQQLRQRPHPHEFTMYYDA 473
          .:*:**:**::**  **** .***:*:*::***************** *****
```
