# Supplementary material for: Differential NtcA Responsiveness to 2-Oxoglutarate Underlies the Diversity of C/N Balance Regulation in Prochlorococcus
Source: Front Microbiol. 2018 Jan 9;8:2641. doi: 10.3389/fmicb.2017.02641 (PMC5767323; doi:10.3389/fmicb.2017.02641)
Supplement: Supplementary Figure 4 — Multiple sequence alignment of ICDH from Prochlorococcus MIT9313, SS120 and MED4. The sequences in FASTA format were taken from CYORF and ClustalW2 from EMBL-EBI was used to make the alignment. (∗) Indicates positions which have a single, fully conserved residue; (:) indicates conservation between groups of strongly similar properties and (.) indicates conservation between groups of weakly similar properties. [file Image4.PDF]

```
MIT9313   MAQFEKLSPPSQGTAIRFVNGQPVIPNEPIIPFIRGDGTGVDIWPATQRVLDAAVTKAYQ 60
SS120     MVHYEKLSPPTTGEKIKFNNGLPIVPANPIIPYIRGDGTGVDIWPATQKVIDQAIEKAYG 60
MED4      MPKFEKLNLPKEGELITFNQGKPNVPNNPIVPYIRGDGTGVDIWPATQLVLDEAIKKSYG 60
          * ::***. *. *  * * :* * :* :**:*:*************** *:* *: *:*

MIT9313   GVRRIEWFKVYAGDEACDLYGTYQYLPEDTLEAIRTYGVAIKGPLTTPIGGGIRSLNVAL 120
SS120     IDRKIEWFKIYAGDEACDLYGTYQYLPKDTIEAIREYGVAIKGPLTTPIGGGIRSLNVAL 120
MED4      DERKINWFKVYAGDEACEIYGTYNYLPQDTIEAIRHFGVAIKGPLTTPIGGGIRSLNVAL 120
            *:*:***:*******::****:***:**:**** :***********************

MIT9313   RQIFDLYSCVRPCRYYKGTPSPHKRPQDLDVIVYRENTEDIYMGVEWEADDPVGKTLREH 180
SS120     RQKFDLYSCVRPCRYYQGTPSPHKHPENLDVIVYRENTEDIYIGIEWESNDPIGIKLIEH 180
MED4      RQIFDLYSCVRPCKYYSGTPSPHKNPQNLDVIVYRENTEDIYMGIEWEADDHTCIDLINH 180
          ** **********:**.*******.*::**************:*:***::*     * :*

MIT9313   LNTVVIPANGKLGKRQIPEGSGIGIKPVSKHGSQRHIRKAIQHALRLKGDKRHVTLVHKG 240
SS120     LNNDVIPASPSLKNRIIPQGSGIGIKPVSKDGSQRHIRRAIQHALKLNGNKRHVTLVHKG 240
MED4      LNNVVIPNSKNLKNRSIPDGSGIGIKPVSKLGSQRHIRKAIEHAKRLSGNKKHVTLVHKG 240
          **. *** . .* :* **:*********** *******:**:** :*.*:*:********

MIT9313   NIMKFTEGAFRDWGYELATSEFRDVCITERESWILGNLENDPQLSIQANARMIEPGYDSL 300
SS120     NIMKFTEGSFRDWGYELATNEFRNECITERESWILSNLEQNPRLSIENNAKLIDPGYESL 300
MED4      NIMKYTEGAFRDWGYELAVNEFRADCITERESWILDNIHKNPEITIENNARKIEPGYDKL 300
          ****:***:*********..***  **********.*:.::*.::*: **: *:***:.*

MIT9313   TPERKASIDAEVHGVLDAIGTSHGNGQWKAMVLVDDRIADSIFQQIQTRPQEYSILATLN 360
SS120     TKEKKDIICNEVQLVINNIHKTHGNNKWKKMVLVDDRIADSIFQQIQTRPQEYSILATLN 360
MED4      TSNKKAFICEEIKEVIASISNSHGNNKWEELIMVDDRIADSIFQQIQTRPQEYSILATLN 360
          * ::*  *  *:: *:  *  .:***.:*: :::***************************

MIT9313   LNGDYISDAAAAMVGGLGMAPGANIGENAAIFEATHGTAPKHAGLDRINPGSVILSGVMM 420
SS120     LNGDYISDAAAAIVGGLGMAPGANIGDRAAIFEATHGTAPKHAGLDRINPGSVILSGVMM 420
MED4      LNGDYVSDAAAAIVGGLGMAPGANIGDNSAIFEATHGTAPKHAGLNKINPGSVILSGVMM 420
          *****:******:*************:.:****************::*************

MIT9313   LEFLGWQEAADLITKGLSAAIANQQVTYDLARLMDPPVDPVSCSGFSEAVISHF 474
SS120     LEYIGWQEAADLITKGLSQSIYDKQVTYDLARLMEPPQSPLSCSEFANAVIERF 474
MED4      LEYFGWNEAAKLVTSGISKAIEEKKVTYDLARLMEPKVAPLSCSGFAEAIISNF 474
          **::**:***.*:*.*:* :* :::*********:*   *:*** *::*:*..*
```
