# Supplementary material for: Differential NtcA Responsiveness to 2-Oxoglutarate Underlies the Diversity of C/N Balance Regulation in Prochlorococcus
Source: Front Microbiol. 2018 Jan 9;8:2641. doi: 10.3389/fmicb.2017.02641 (PMC5767323; doi:10.3389/fmicb.2017.02641)
Supplement: Supplementary Table 1 — Oligonucleotides used for cloning and qRT-PCR. [file Table1.DOCX]

**Supplementary Table 1. Oligonucleotides used for cloning and qRT-PCR.**

**Oligonucleotides used for cloning**

The oligonucleotides used for amplifying *ntcA* from *Prochlorococcus* are shown in the following sections. The optimal Tm of each pair of primers was selected doing a temperature gradient PCR.

**** ntcA* MIT9313**

| Oligonucleotides | Sequence 5´- 3´ | Function |
| --- | --- | --- |
| FNTC | TCATATTTCTGCCGTTTGCT | Amplification of *ntcA* (796 bp) |
| RNTC | AACCCGTTTCACCATCCT |  |
| FNNM2 | CATATGATGGCCAACTCGCCTGCT | Introduction of NdeI-BamHI restriction sites |
| RBNM2 | GGATCCTCAGTTGAATTTTTTAGCAAGGGC |  |

**** ntcA* SS120**

| Oligonucleotides | Sequence 5´- 3´ | Function |
| --- | --- | --- |
| FSNTC1 | AATCTAGCGGGACTTGGAA | Amplification of *ntcA* (1036 bp) |
| RSNTC1 | CCTCCCAAAAGAAGCAAAA |  |
| FNNS2 | CATATGATGACAGGTTCTGCTAACTC | Introduction of NdeI-BamHI restriction sites |
| RBNS2 | GGATCCTTAGTTAAAACGTTTAGCAAG |  |

**** ntcA* MED4**

| Oligonucleotides | Sequence 5´- 3´ | Function |
| --- | --- | --- |
| PMM0246-1 | CATATGTCACCTGCTTCTAGAGGATT | Amplification of *ntcA* (816 bp) introducing NdeI-BamHI restriction sites |
| PMM0246-2 | GGATCCGAAAAGCCAACCAGACAC |  |

The following table shows the oligonucleotides that were used for checking the ligation and for sequencing each step during the cloning process. SP6 and T7 were used for checking pGEM-T and pSPARK vectors and T7/T7 terminator for checking pET-15b.

**Generic oligonucleotides**

| Oligonucleotides | Sequence 5´- 3´ |
| --- | --- |
| T7 | TAATACGACTCACTATAGGG |
| SP6 | TATTTAGGTGACACTTATAG |
| T7 Terminator | GCTAGTTATTGCTCAGCGG |

**Oligonucleotides used for qRT-PCR**

The Tm for oligonucleotides for RT-PCR was 58 ºC. All the oligonucleotides used are shown in the following sections.

***Prochlorococcus* SS120**

| Oligonucleotides | Sequence 5´- 3´ | Gene |
| --- | --- | --- |
| FALL-1 | AGCTCCTGCTGGCTCAGTTA | *ntcA* |
| RALL-1 | GAGAAGTAGCCCAACCCCAC |  |
| SRF | CTCTCGGTTGAGGAAAGTC | *rnpB* |
| SRR | CCTTGCCTGTGCTCTATG |  |

***Prochlorococcus* MIT9313**

| Oligonucleotides | Sequence 5´- 3´ | Gene |
| --- | --- | --- |
| RT-FTCA | GCGTGGGGTTACTTCTGC | *ntcA* |
| RT-RTCA | CGATGGACTGATGTGAGAGG |  |
| FRPM | CTATCTAGGACCGCCGTTAC | *rnpB* |
| RRPM | GAGAGTGCCACAGAAAAACA |  |

***Prochlorococcus* MED4**

| Oligonucleotides | Sequence 5´- 3´ | Gene |
| --- | --- | --- |
| RT-FNP | CCTGGAGATCCTGCTGAAAG | *ntcA* |
| RT-RNP | CAATTGCTCTCAAAACAGAA |  |
| FE | ACAGAAACATACCGCCTAAT | *rnpB* |
| RE | ACCTAGCCAACACTTCTCAA |  |
